# Identification of Novel JAK2 Inhibitors from Amino Derivatives of Epoxyalantolactone: In Silico and In Vitro Studies

**DOI:** 10.3390/ijms27010329

**Published:** 2025-12-28

**Authors:** Duangjai Todsaporn, Kamonpan Sanachai, Chanat Aonbangkhen, Rungtiva P. Poo-arporn, Victor Kartsev, Sergey Pukhov, Svetlana Afanasyeva, Athina Geronikaki, Thanyada Rungrotmongkol

**Affiliations:** 1 Center of Excellence in Structural and Computational Biology, Department of Biochemistry, Faculty of Science, Chulalongkorn University, Bangkok 10330, Thailand; fai.dt8@gmail.com; 2 Department of Biochemistry, Faculty of Science, Khon Kaen University, Khon Kaen 40002, Thailand; kamosa@kku.ac.th; 3 Center of Excellence in Natural Products Chemistry (CENP), Department of Chemistry, Faculty of Science, Chulalongkorn University, Bangkok 10330, Thailand; chanat.a@chula.ac.th; 4 Biological Engineering Program, Faculty of Engineering, King Mongkut’s University of Technology Thonburi, Bangkok 10140, Thailand; rungtiva.pal@kmutt.ac.th; 5 InterBioScreen, Chernogolovka 142432, Russia; vkartsev@ibscreen.chg.ru; 6 Institute of Physiologically Active Compounds, Federal Research Center of Problems of Chemical Physics and Medicinal Chemistry, Russian Academy of Sciences, Chernogolovka 142432, Russia; serbg2007@yandex.ru (S.P.); svetlana.afanasyeva@yandex.ru (S.A.); 7 Department of Pharmaceutical Chemistry, School of Pharmacy, Aristotle University of Thessaloniki, 54124 Thessaloniki, Greece; 8 Program in Bioinformatics and Computational Biology, Graduate School, Chulalongkorn University, Bangkok 10330, Thailand

**Keywords:** JAK inhibitor, sesquiterpene lactone-based scaffolds, lead optimization, kinase-targeted anticancer agents, molecular docking, MD simulations

## Abstract

Janus kinase 2 (JAK2) is a key mediator of oncogenic signaling and a promising therapeutic target in cervical cancer. This study employed a combination of in silico and in vitro approach to discover sesquiterpene lactone (**SL**) derivatives with JAK2 inhibitory activity. Molecular docking of forty SL derivatives, followed by drug-likeness and toxicity prediction, led to the selection of six candidates for synthesis and biological evaluation. Among these, **SL10** (12.7 nM) and **SL35** (21.7 nM) demonstrated potent JAK2 inhibition and exhibited selective cytotoxicity toward HeLa cervical cancer cells, outperforming ruxolitinib. Flow cytometry confirmed apoptosis induction and ROS elevation, suggesting ROS-mediated cytotoxic mechanisms. The 1 µs MD simulations demonstrated that both hydrogen bonding and hydrophobic interactions are critical determinants in stabilizing potent **SL**s–JAK2 complexes. These findings support **SL10** and **SL35** as promising scaffolds for further development of JAK2-targeted therapies in cervical cancer.

## 1. Introduction

Cervical cancer remains a pressing global health concern, ranking as the fourth most common malignancy in women, with an estimated 660,000 new cases and 350,000 deaths reported in 2022 [1]. Persistent infection with high-risk human papillomaviruses (HPVs), primarily HPV16 and HPV18, and to a lesser extent HPV31, HPV33, and HPV45, accounts for the majority of invasive cases [2]. The oncogenic potential of these viruses is attributed to the E6 and E7 oncoproteins, which disable essential tumor suppressors and promote malignant transformation [3]. The disease disproportionately affects women in low- and middle-income countries, where it imposes a considerable burden on public health systems. Although HPV vaccination is the most reliable method for primary prevention, and cytology-based screening such as the Pap test has improved early detection, these strategies have limitations. The Pap test functions only as a screening tool rather than a diagnostic procedure; it cannot assess lesion size or invasion depth, and cytologic changes may be subtle, leading to false negatives [4] and delayed diagnosis [5]. As a result, despite the progress of screening programs, cervical cancer continues to contribute significantly to cancer mortality, largely due to late-stage diagnosis. Since 2006, three prophylactic vaccines against oncogenic HPV have been introduced in high-income countries: Gardasil^®^ (quadrivalent, HPV6/11/16/18) [6], Cervarix^®^ (bivalent, HPV16/18) [7], and Gardasil 9^®^ (nonavalent, HPV6/11/16/18/31/33/45/52/58) [8], approved in 2006, 2007, and 2014, respectively. These vaccines have markedly reduced the prevalence of HPV infection and HPV-related conditions, including genital warts, precancerous lesions, and cervical cancer. Current management of early-stage disease relies primarily on surgery, often in combination with radiotherapy or chemotherapy [9]. Each of these modalities presents limitations: surgery can compromise fertility, radiotherapy is restricted to non-metastatic disease, and chemotherapy is associated with systemic toxicity, poor pharmacokinetics, and rapid acquisition of drug resistance through mechanisms such as apoptosis evasion, enhanced DNA repair, and reduced drug uptake [10,11]. Consequently, there is a critical need for therapies that are both more effective and more selective in the treatment of cervical cancer. Compared with conventional chemotherapy, which is limited by toxicity and resistance, targeted therapies provide distinct advantages, including greater efficacy, reduced off-target effects, and the potential to improve patients’ overall quality of life.

One promising molecular target for cervical cancer therapy is Janus kinase 2 (JAK2), a member of the non-receptor protein tyrosine kinase family. The JAK family comprises four isoforms: JAK1, JAK2, JAK3, and TYK2 [12]. These enzymes are crucial components of the JAK/STAT pathway, which becomes activated by cytokines, initiating a cascade of signals vital for the development or homeostasis of an organism [13,14]. The JAK kinases serve as intermediaries between a cytokine signal and the phosphorylated transcription factor STAT. A JAK cytokine receptor consists of multiple subunits, with certain chains associated with a specific JAK isozyme [15]. JAKs are ubiquitous in virtually all cell types. JAK1 and JAK2 exhibit diverse functions, including hematopoiesis, growth, and neural development, while JAK3 and TYK2 primarily regulate the immune response [16]. Hence, aberrant JAK/STAT signaling may lead to a various of diseases, including cancer, inflammation, and autoimmune disorders [15,16,17]. Among the JAK family, JAK2 represents a significant target for cancer treatment owing to its involvement in cell growth and survival [18]. The occurrence of the JAK2 V617 mutation, commonly found in Philadelphia chromosome-negative myeloproliferative neoplasms (MPNs), has driven the development of JAK2 inhibitors [19,20]. Nevertheless, a major challenge in the development of a targeted JAK2 inhibitor lies in the similarity of the ATP binding site across members of the JAK family [21,22,23]. The current approved JAK inhibitors including Ruxolitinib, Tofacitinib, Baricitinib, and Oclacitinib, which selectively target JAK1/2, JAK1/3, JAK1/2, and JAK1, respectively [24,25,26]. The application of these inhibitors targeting the JAK family has led to successful treatment outcomes for individuals suffering from myelofibrosis, polycythemia vera, rheumatoid arthritis, pruritus, or MPN [27]. However, both Ruxolitinib and Tofacitinib exhibit off-target kinase interactions, potentially resulting in undesirable side effects [25,28]. Consequently, there remains a substantial demand for a JAK2-specific inhibitor. In solid tumors, JAK2 signaling has an essential role in colorectal cancer, breast cancer, lung cancer, prostate cancer, and cervical cancer [29,30]. Morgan E. L. et al. reported that Ruxolitinib, an available JAK2 inhibitor, inhibits JAK2 and induces apoptosis in cervical cancer cell lines HeLa and CaSKi. Furthermore, they found that JAK2 phosphorylation correlates with both STAT3 and STAT5 phosphorylation, highlighting JAK2/STAT3 and JAK2/STAT5 are functional in cervical cancer cell [31]. Previous studies have shown that selenadiazole can suppress tumor growth in cervical cancer cells by elevating intracellular ROS levels and triggering mitochondrial apoptosis through modulation of the JAK2/STAT3 signaling pathway [32]. These findings indicate that a JAK2 inhibitor may be a valuable target for the treatment of cervical cancer.

Sesquiterpene lactones (**SL**s) are a class of plant-derived secondary metabolites predominantly found in the Asteraceae family, known for their broad pharmacological potential [33]. **SL**s have demonstrated a wide range of biological activities, including antioxidant [34,35], immunomodulatory [36,37], antitumor [38,39], antibacterial, antifungal [40,41], anti-inflammatory [35,42], antiparasitic [43], antiviral [44,45], and antidiabetic [46] properties. Several reviews have summarized the anticancer and skin-related effects of **SL**s [47], while Fateh et al. further explored their role in cellular differentiation [48]. Keeratichamroen et al. reported that three **SL**s isolated from *Gymnanthemum extensum*—vernodalin (VDa), vernolepin (VLe), and vernolide (VLi)—exerted dose-dependent cytostatic and cytotoxic effects against A549 lung cancer cells by modulating the JAK2/STAT3 pathway [49]. Previous studies have demonstrated a subset of amino derivatives of epoxyalantolactone was reported to exhibit significant antineoplastic effects [50]. Inspired by these observations, the present study aims to conduct a systematic investigation into the biological properties of a previously uncharacterized set of amino-substituted epoxyalantolactone derivatives, featuring structural variations at the R^1^ and R^2^ positions. This study focuses on assessing their potential as novel Janus kinase 2 (JAK2) inhibitors, a therapeutically relevant target known to play a critical role in oncogenic signaling pathways.

**Figure 1:**
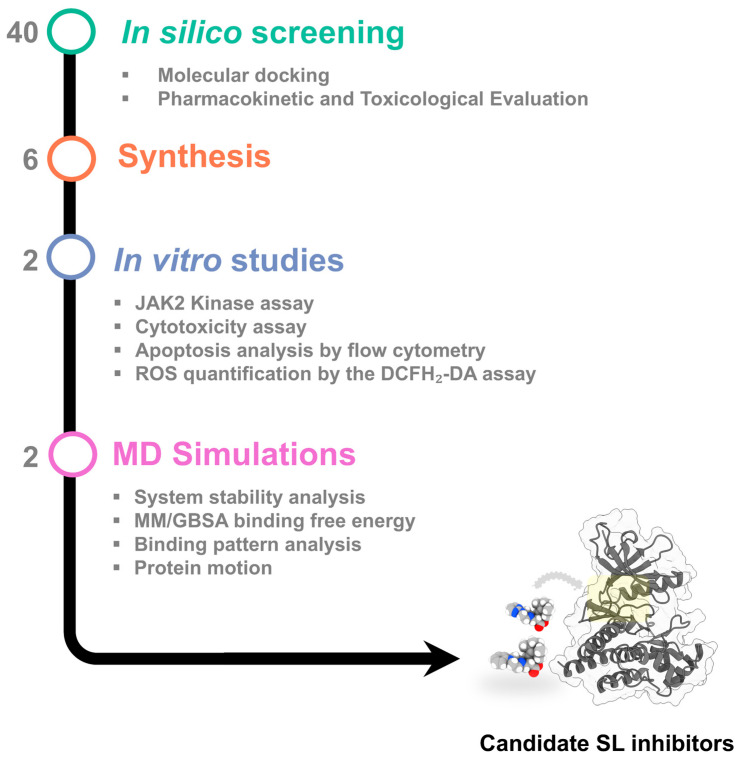
Workflow for the identification of candidate JAK2-targeting sesquiterpene lactone (**SL**) inhibitors. (i) In silico screening of 40 **SL** derivatives using molecular docking, followed by pharmacokinetic and toxicological evaluation. (ii) Synthesis of top-ranked compounds for biological testing. (iii) In vitro studies comprising JAK2 kinase inhibition, cytotoxicity assays, apoptosis analysis by flow cytometry and ROS quantification by DCFH_2_-DA assay (iv) Molecular dynamics simulations to evaluate system stability, binding free energy calculation, interaction profiles and protein motion of the candidate compounds.

Computational techniques such as virtual screening have revolutionized early-stage drug discovery by efficiently identifying promising candidates with optimal pharmacological profiles [51,52]. Molecular docking enables rapid estimation of binding affinity, while molecular dynamics (MD) simulations provide insights into the dynamic behavior and stability of ligand–target interactions [53,54,55,56,57]. In this study, a structure-based virtual screening approach was employed to identify sesquiterpene lactones with potent JAK2 inhibitory potential. Promising candidates were experimentally validated using a combination of enzymatic and cell-based assays, including kinase inhibition, cytotoxicity, ROS generation, and apoptosis analysis via flow cytometry in HPV-positive cervical cancer cells. Finally, MD simulations were performed to explore the structural stability and binding interactions of top hits (Figure 1). This integrated strategy highlights the utility of combining computational and experimental approaches in accelerating the development of targeted therapies for cervical cancer.

## 2. Results and Discussion

### 2.1. In Silico Evaluation of Compound Binding Affinity, Pharmacokinetics, and Safety Profiles

**Figure 2:**
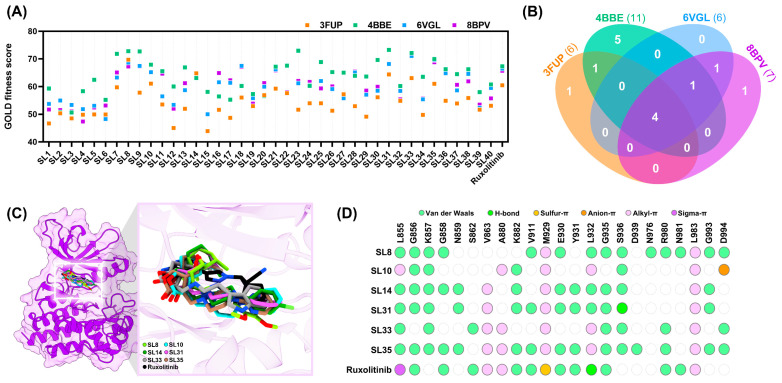
Molecular docking analysis of sesquiterpene lactone (**SL**) derivatives targeting JAK2 kinase. (**A**) GOLD fitness scores of 40 **SL** derivatives and ruxolitinib docked against four JAK2 crystal structures (PDB IDs: 3FUP, 4BBE, 6VGL, and 8BPV). (**B**) Venn diagram summarizing the overlapping top-scoring compounds identified from the four JAK2 crystal structures. (**C**) Superimposition of ruxolitinib and selected **SL** derivatives within the ATP-binding site of JAK2 (based on PDB ID: 3FUP). (**D**) Protein–ligand interaction profiles generated using Discovery Studio, highlighting interaction types including van der Waals forces, hydrogen bonds, π interactions, and electrostatic contacts in comparison with ruxolitinib.

The binding affinity of 40 sesquiterpene lactone (**SL**) derivatives toward four crystal structures of JAK2 kinase (PDB IDs: 3FUP, 4BBE, 6VGL, and 8BPV) was evaluated via molecular docking using the GOLD program, with the two-dimensional structures of these compounds provided in Figure S1. All **SL** derivatives successfully docked into the ATP-binding pocket of JAK2, and their respective GOLD fitness scores are presented in Figure 2A. The clinically approved inhibitor ruxolitinib was employed as a reference compound, yielding GOLD fitness scores of 60.48, 67.35, 66.49, and 65.73 for 3FUP, 4BBE, 6VGL, and 8BPV, respectively. The **SL** derivatives exhibited fitness scores ranging from 43.91 to 64.83 (3FUP), 50.64 to 73.30 (4BBE), 48.29 to 71.10 (6VGL), and 47.37 to 72.22 (8BPV). Notably, a subset of **SL** compounds outperformed ruxolitinib, comprising 6 derivatives in the 3FUP structure, 11 in 4BBE, 6 in 6VGL, and 7 in 8BPV structure. Given the consistent predictive trend of docking results across the four structures, the six highest-scoring **SL** derivatives from the 3FUP model were selected for further analysis.

Structural superimposition analysis (Figure 2C) revealed that the selected **SL** derivatives aligned near the hinge region of the JAK2 active site in a manner similar to ruxolitinib. Interaction profile analysis (Figure 2D) demonstrated that these compounds predominantly engaged in extensive van der Waals interactions with critical residues, including L855, G856, K857, V911, E930, L932, G935, S936, N976, N981, G993, and D994. Moreover, characteristic alkyl–π interactions involving residues V863 and A880 within the glycine-rich loop (G-loop), as well as residues M929, L932, and L983 at the hinge region, further stabilized the ligand–protein complexes. Collectively, the observed interaction patterns closely resembled those established by ruxolitinib, underscoring the promising potential of these **SL** derivatives as effective JAK2 inhibitors.

The drug-likeness of the screened **SL** compounds was assessed based on Lipinski’s Rule of Five, with physicochemical parameters summarized in Table 1**.** All six compounds complied with the rule, suggesting favorable oral bioavailability profiles. Furthermore, the safety profiles of the screened **SL** compounds were investigated using the ProTox 3.0 platform, with predictions focused on two principal categories: (i) organ-specific toxicities, including hepatotoxicity, nephrotoxicity, cardiotoxicity, and neurotoxicity, and (ii) toxicity endpoints such as mutagenicity and cytotoxicity [58] (Table S1). Compounds were classified as either “inactive” (non-toxic) or “active” (toxic) based on their predicted properties and median lethal dose (LD_50_, mg/kg), following established toxicity standards. None of the screened compounds were predicted to exhibit hepatotoxicity, cardiotoxicity, mutagenicity, or cytotoxicity. **SL14** was the only compound associated with potential nephrotoxicity. Neurotoxic potential was observed in all compounds except **SL8**. This finding may be clinically relevant in the context of cervical cancer, where neurotoxicity has been reported in patients undergoing chemoradiotherapy, potentially exacerbated by psychological stress [59]. According to the Globally Harmonized System of Classification and Labeling of Chemicals (GHS) [60], substances are categorized from Class 1 (most toxic) to Class 5 (least harmful). All six **SL** compounds demonstrated predicted LD_50_ values ranging from 700 to 2000 mg/kg, placing them within GHS toxicity Class 4. This classification indicates relatively low acute toxicity and supports their further development from a safety perspective. Altogether, these findings support the pharmacological potential and safety of the selected compounds. The six **SL** derivatives with the most favorable profiles were synthesized and subjected to subsequent experimental validation.

### 2.2. Chemistry

Natural sesquiterpene lactones containing an exocyclic methylene group at the β-position of the lactone ring can readily react with N-nucleophiles, particularly amines [61]. These reactions proceed selectively without interference from other functional moieties, leading to the generation of amino derivatives [62,63]. Such compounds are reported to display significant biological properties, especially antitumor potential, making them attractive scaffolds for drug discovery [64].

**Scheme 1:**
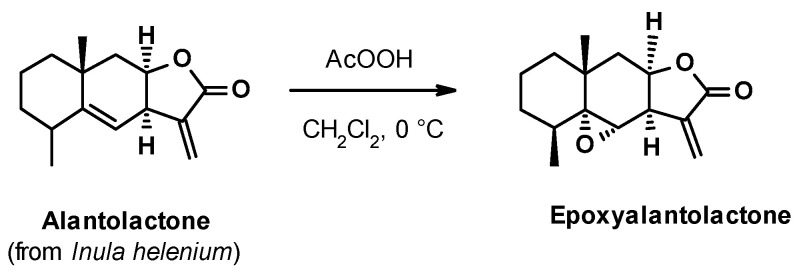
Epoxidation of alantolactone.

Epoxidation of alantolactone at the nonconjugated double bond was performed using a previously described method [65,66]. The reactions proceeded at room temperature over 24 h. The process afforded the epoxy derivative in quantitative yield, as depicted in Scheme 1.

**Scheme 2:**
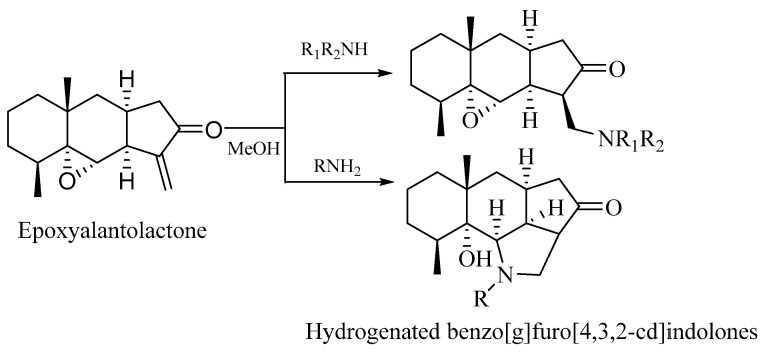
Reaction of epoxyalantolactone with amines.

Epoxyalantolactone contains an α-methylene-γ-lactone fragment that participates in Michael addition with primary or secondary amines. To obtain analogs with improved biological potential over epoxyalantolactone, a panel of pharmacophoric amines was selected (3,4-dimethoxybenzylamine, 1-phenylpiperazine, 1-(4-fluorophenyl)piperazine, 1-(m-tolyl)piperazine, 2-piperazin-1-yl-pyrimidine and 3-(2-aminoethyl)indole (tryptamine)). When reacted with secondary amines under mild conditions (equimolar ratios in methanol, room temperature), the process was both regio- and stereoselective, consistently affording a single stereoisomer in high yield that typically precipitated from the mixture. When primary amines were introduced into the reaction, products of an unusual heterocyclic system were obtained (**SL8** and **SL33**) (Scheme 2).

### 2.3. In Vitro Validation of JAK2 Inhibitory Activity

**Figure 3:**
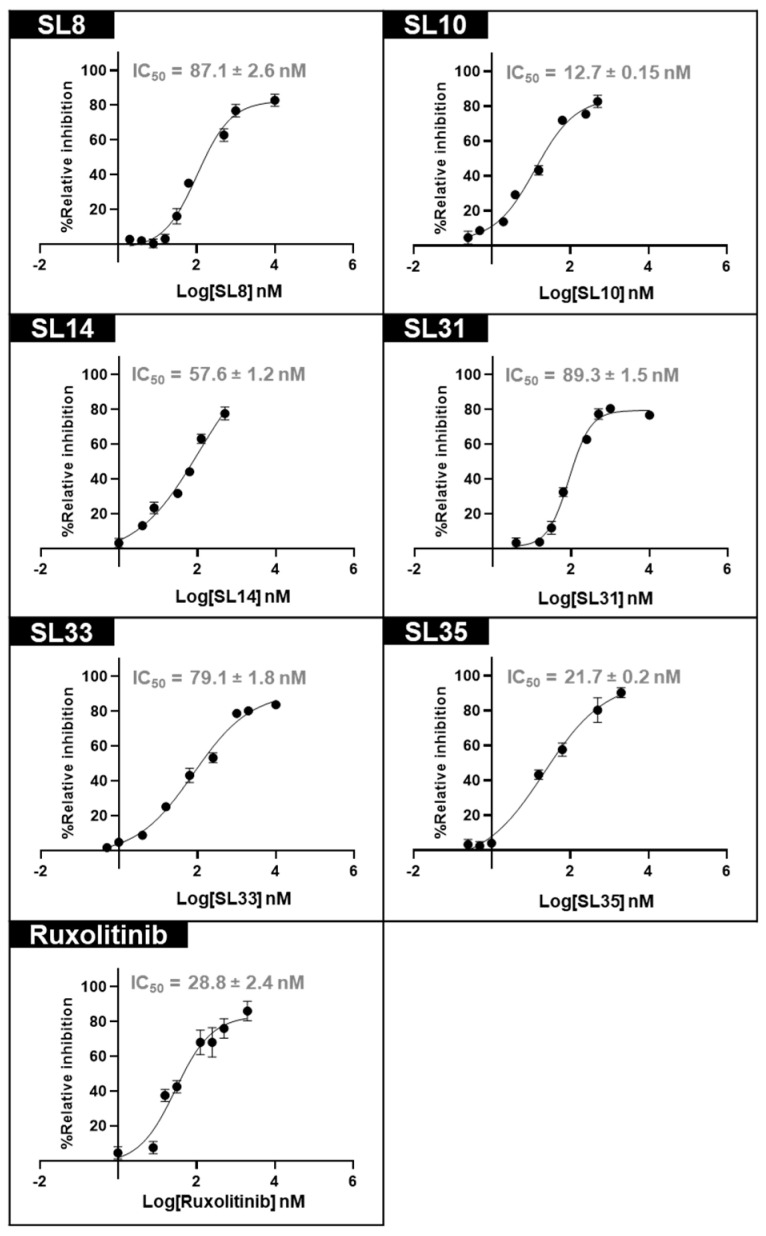
Dose–response curves and IC_50_ values of the screened **SL** derivatives and ruxolitinib obtained from JAK2 kinase inhibition assays. Data represent mean ± SEM from three independent experiments.

In light of the molecular docking and pharmacokinetic/toxicological evaluations, six **SL** derivatives were selected as promising JAK2 inhibitor candidates. To confirm their inhibitory activity, an in vitro JAK2 kinase assay was performed using ruxolitinib as the reference compound (IC_50_ = 28.8 ± 2.4 nM). As shown in Figure 3, all six compounds demonstrated inhibitory activity in the nanomolar range. The strongest inhibition was observed for **SL10** (IC_50_ = 12.7 ± 0.15 nM) and **SL35** (IC_50_ = 21.7 ± 0.2 nM), which contain phenyl-piperazine and pyrimidinyl-piperazine groups, respectively. These findings suggest that bulky piperazine moieties linked to aromatic or heteroaromatic rings are favorable for potency within this scaffold. **SL14**, bearing a fluorophenyl-piperazine group, exhibited moderate activity (IC_50_ = 57.6 ± 1.2 nM). In contrast, compounds with smaller or less complex substituents, such as **SL8** with a dimethoxyphenethylamine group (IC_50_ = 87.1 ± 2.6 nM), **SL31** with a methylphenyl-piperazine group (IC_50_ = 89.3 ± 1.5 nM), and **SL33** with an indolylethylamine group (IC_50_ = 79.1 ± 1.8 nM), showed markedly weaker inhibition. the results highlight that bulky aryl- or heteroaryl-piperazine groups are more favorable for JAK2 inhibition than smaller substituents. Notably, **SL10** and **SL35** outperformed several previously reported JAK2 inhibitors, including the 4-piperazinyl-2-aminopyrimidine derivative (IC_50_ = 27 nM) [67], thiazole aromatic alkylamino analogs (IC_50_ = 17.6–20.3 nM) [53], purine-2,6-diamine derivative (IC_50_ = 22 nM) [62], 6-diamino-substituted pyrimidine derivatives (IC_50_ = 200 µM) [68], and pyrazole derivatives (IC_50_ = 94 nM) [69]. These findings emphasize **SL10** and **SL35** as promising candidates for further development as potent JAK2 inhibitors.

Cross-docking represents a valuable approach for addressing selectivity and has been successfully applied in previous studies to validate selectivity toward JAK proteins [70,71,72]. To investigate the selectivity profile of the screened sesquiterpene lactone derivatives, a cross-docking approach was performed against other members of the JAK family, including JAK1 (PDB ID: 6N7D), JAK2 (PDB ID: 3FUP), JAK3 (PDB ID: 5TOZ), and TYK2 (PDB ID: 4GJ2). As shown in Table S2, ruxolitinib, an approved JAK1/2 inhibitor, showed comparable GOLD fitness scores for JAK1 (58.58) and JAK2 (60.48), but notably weak interaction for JAK3 (52.46) and TYK2 (53.26), in agreement with its reported JAK1/2 selectivity [73]. Among the six screened compounds, **SL1**4, **SL31**, **SL33**, and **SL35** achieved the high fitness scores for both JAK1 (59.90–65.31) and JAK2 (61.03–64.83), with ≤1 unit differences, indicating potential dual JAK1/2 inhibition. In contrast, **SL8** and **SL10** displayed their highest fitness score for JAK2, with 61.73 and 61.09, respectively, and markedly lower fitness score for other kinases. Cross-docking analysis revealed a clear preference of the screened compounds for JAK2 over other JAK family kinases, supporting their potential as selective inhibitors with reduced off-target interactions. Furthermore, in silico kinase profiling was conducted using the KinomeX platform (Figure S3 and Table S4). At a probability of cut-off of 0.7, both **SL10** and **SL35** showed predicted probabilities for JAK2 above 0.8, consistent with their design as JAK2 inhibitors. In addition, **SL10** was predicted to interact with 30 kinases and **SL35** with 23 kinases above the threshold, suggesting a relatively selective profile but not excluding possible cross-reactivity. For comparison, ruxolitinib also demonstrated strong predicted activity toward JAK2 but was associated with a broader interaction spectrum involving 41 kinases, in agreement with its known off-target liabilities in clinical use. These results indicate that **SL10** and **SL35** may possess improved selectivity compared with ruxolitinib, although this conclusion remains subject to the inherent limitations of in silico prediction. Collectively, the cross-docking analyses and kinome profiling suggest that **SL10** and **SL35** may display a preference for JAK2 binding. Nonetheless, these computational results should be interpreted with caution, as they do not constitute definitive biochemical evidence of selectivity. Comprehensive kinase panel profiling will be required to substantiate this potential trend, delineate possible off-target interactions, and provide a more robust evaluation of the selectivity and safety profiles of **SL10** and **SL35** as prospective JAK2-directed therapeutic candidates.

### 2.4. In Vitro Study of Anti-Proliferative Effects of SL Compounds

**Figure 4:**
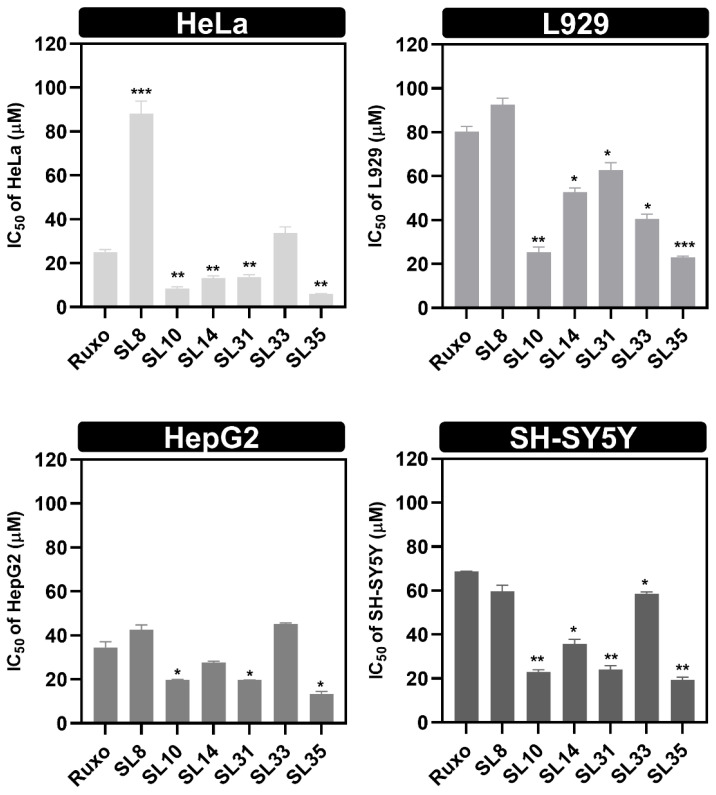
IC_50_ values of six sesquiterpene lactone (**SL**) derivatives and ruxolitinib (Ruxo) were determined in human cervical adenocarcinoma (HeLa), mouse fibroblast (L929), human hepatocellular carcinoma (HepG2) and human neuroblastoma (SH-SY5Y) cell lines using the MTT assay. Data are presented as mean ± SD from three independent experiments. Statistical analysis was performed using one-way ANOVA (*p < 0.05, **p < 0.01, *** *p* < 0.001, compared to ruxolitinib).

Given their promising JAK2 inhibitory potential, six sesquiterpene lactone derivatives (**SL8**, **SL10**, **SL14**, **SL31**, **SL33**, and **SL35**) were evaluated for their anti-proliferative activity in HeLa cervical cancer cells using the MTT assay, with ruxolitinib (Ruxo) included as a reference drug (Figure 4 and Table S3). The **SL** derivatives displayed variable cytotoxic potency, with IC_50_ values ranging from 6.07 ± 0.11 to 88.2 ± 5.7 μM. Among them, **SL10** (IC_50_ = 8.47 ± 0.74 μM) and **SL35** (IC_50_ = 6.07 ± 0.11 μM) exerted the strongest inhibitory effects, surpassing ruxolitinib (IC_50_ = 25.0 ± 1.3 μM). **SL14** (IC_50_ = 13.2 ± 1.1 μM) and **SL31** (IC_50_ = 13.7 ± 1.0 μM) exhibited moderate activity, whereas **SL8** (IC_50_ = 88.2 ± 5.7 μM) and **SL33** (IC_50_ = 33.8 ± 2.8 μM) were comparatively less active. These results indicate that structural modifications on the **SL** scaffold markedly influence cytotoxic potency.

To determine whether the cytotoxic effects were specific to malignant cells or reflected general toxicity, the compounds were evaluated in L929 fibroblasts, a representative model of non-cancerous proliferative cells. **SL10** and **SL35** showed three- to four-fold higher IC_50_ values in L929 (25.4 ± 2.4 μM and 23.1 ± 0.5 μM, respectively) compared to HeLa, supporting selective anticancer activity. By contrast, **SL8** and **SL33** retained high IC_50_ values in both HeLa and L929, indicating poor selectivity.

Since toxicity prediction suggested potential risks of hepatotoxicity and nephrotoxicity (Table S2), the compounds were further assessed in HepG2 hepatocytes and SH-SY5Y neuroblastoma cells to confirm their safety profiles. These cell lines have been widely employed in previous studies as in vitro models for evaluating hepatotoxic and neurotoxic responses, supporting their suitability for preliminary toxicity assessment [74,75,76,77]. The results revealed that **SL10** (IC_50_ = 19.7 ± 0.3 μM in HepG2 and 23.1 ± 0.9 μM in SH-SY5Y) and **SL35** (13.3 ± 1.1 and 19.3 ± 1.4, respectively) displayed relatively low toxicity across liver and neuronal models when compared to their strong activity in HeLa cells (8.47 and 6.07 μM, respectively), indicating a favorable therapeutic margin. **SL14** and **SL31** exhibited intermediate profiles, suggesting some degree of HepG2 and SH-SY5Y at higher concentrations. In contrast, **SL8** and **SL33** demonstrated broad cytotoxicity with poor selectivity, showing significant inhibitory effects not only in HeLa but also in liver and neuronal cells. These findings highlight **SL10** and **SL35** as the most promising candidates with reduced risk of systemic toxicity, whereas **SL8** and **SL33** appear to exert broad-spectrum toxicity across multiple tissues. Nonetheless, because the MTT assay reflects metabolic activity rather than direct cell viability, a single assay cannot confirm selective anti-proliferative effects. Additional cytotoxicity measurements would be required to substantiate these findings. Although the present study focused primarily on JAK2 inhibition and cytotoxic activity, the downstream effects on STAT3 and STAT5 phosphorylation remain an important aspect that should be further investigated clarifying the mechanisms of **SL10** and **SL35**.

Based on both enzyme-based and cell-based assay results, **SL10** and **SL35** emerged as the most promising candidates, exhibiting potent JAK2 inhibition, selective anti-proliferative activity against cervical cancer cells, minimal toxicity toward normal cells, and superior efficacy compared to ruxolitinib. These two compounds were therefore selected for further investigation, including evaluation of their apoptosis-inducing potential in cancer cells, assessment of intracellular reactive oxygen species (ROS) levels and characterization of binding pattern of JAK2/potent **SL**s through all-atom MD simulations.

### 2.5. Evaluation of Apoptosis Induced by Lead Compounds in Cancer Cells

**Figure 5:**
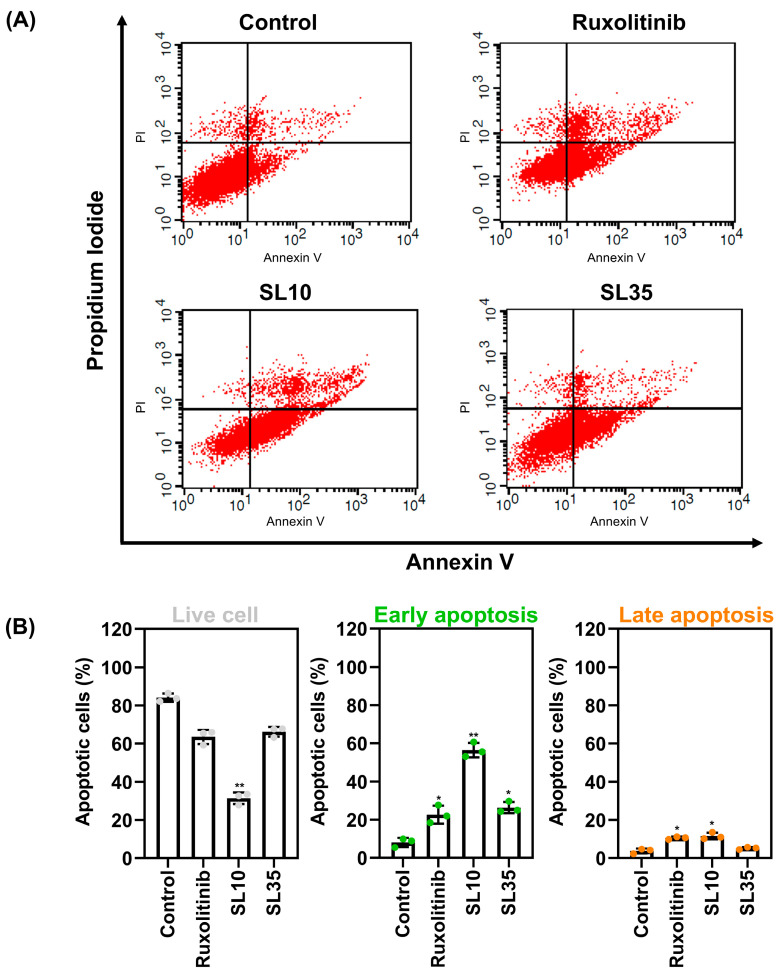
Apoptosis induction by **SL10** and **SL35** in HeLa cells following 48 h treatment. (**A**) Representative dot plots from flow cytometry analysis using Annexin V-FITC/propidium iodide (PI) dual staining to distinguish live cells (**lower left**), early apoptotic cells (**lower right**), late apoptotic cells (**upper right**), and necrotic cells (**upper left**). (**B**) Quantification of live cells, early apoptotic cells, and late apoptotic cells following treatment with ruxolitinib, **SL10**, and **SL35**. Data are presented as mean ± SD from three independent experiments. Statistical analysis was performed using one-way ANOVA (* *p* < 0.05 and ** *p* < 0.01 compared to vehicle control (0.1% DMSO).

To investigate whether the anti-proliferative effects of **SL10** and **SL35** were associated with apoptosis induction, Annexin V-FITC/PI dual staining followed by flow cytometry was performed in HeLa cells after 48 h of treatment. As shown in Figure 5 and Figure S2, the majority of cells in the control group remained viable (84.03 ± 1.25%), with low levels of early (8.10 ± 1.38%) and late (3.73 ± 0.74%) apoptosis.

Treatment with ruxolitinib significantly decreased the proportion of viable cells to 63.49 ± 2.17% and increased both early (22.65 ± 2.71%) and late (10.61 ± 0.40%) apoptosis, confirming its pro-apoptotic activity. Notably, **SL10** demonstrated the most pronounced apoptotic effect, reducing cell viability to 31.30 ± 2.21% and markedly increasing early and late apoptosis to 56.39 ± 2.21% and 11.61 ± 0.99%, respectively, surpassing the effect of ruxolitinib. In comparison, **SL35** induced moderate apoptosis, with 66.19 ± 1.51% viable cells, and increased early (26.30 ± 1.69%) and late apoptosis (5.20 ± 0.34%). These findings indicate that both **SL10** and **SL35** promote apoptosis in HeLa cells, with **SL10** exhibiting superior potency. These findings indicate that both **SL10** and **SL35** promote apoptosis in HeLa cells, with **SL10** exhibiting superior potency. This observation is consistent with previous studies reporting that various sesquiterpene lactone derivatives can induce apoptosis in HeLa cells [78,79].

### 2.6. Investigation of Intracellular ROS Generation Induced by Lead Compounds

**Figure 6:**
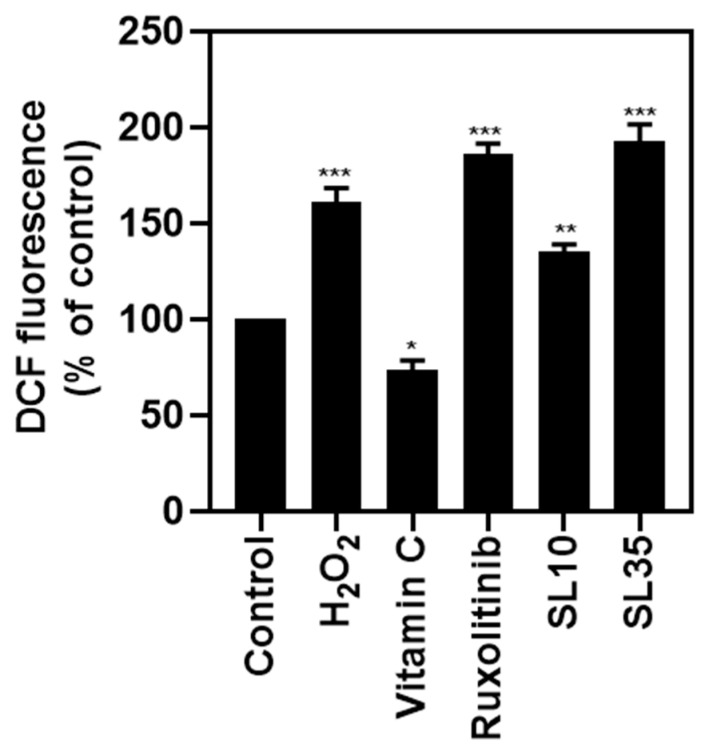
Effect of lead compounds on intracellular ROS generation in HeLa cells. HeLa cells were treated with lead compounds (**SL10** and **SL35**), H_2_O_2_ (positive control), Vitamin C (negative control), or Ruxolitinib for 24 h. Intracellular ROS levels were measured using the DCFH-DA assay and expressed as DCF fluorescence (% of control). Data are presented as mean ± SD (n = 3). Statistical significance is indicated as * *p* < 0.05, ** *p* < 0.01, and *** *p* < 0.001 relative to the control.

The potential involvement of intracellular ROS generation in the cytotoxic and apoptosis-inducing activities of **SL10** and **SL35** were subsequently examined in HeLa cells. As shown in Figure 6, treatment with H_2_O_2_, used as a positive control, significantly increased intracellular ROS levels by approximately 1.7-fold compared to the vehicle control. Conversely, Vitamin C, employed as a negative control, reduced ROS levels to about 0.8-fold relative to the control. Ruxolitinib significantly increased intracellular ROS levels to approximately 1.9-fold compared to the control, while **SL35** induced a comparable elevation to around 2.0-fold. **SL10** also promoted a moderate but statistically significant rise in ROS levels, reaching approximately 1.4-fold relative to the control. These results confirm that both **SL10** and **SL35** effectively enhance intracellular ROS production in HeLa cells. Consistent with these observations, intracellular ROS accumulation is recognized as an effective strategy for selectively inducing cancer cell death, given the higher oxidative stress typically observed in cancer cells compared to normal cells [80]. Sesquiterpene lactone compounds, including alantolactone [81], ambrosin [82], brevilin A [83], britannin [84], lactucopicrin [85], and helenalin [86], have been reported to promote apoptosis through ROS generation in various cancer cell types. These observations align with evidence that selenadiazole drives ROS-dependent mitochondrial apoptosis via JAK2/STAT3 signaling, suggesting that **SL10** and **SL35** may increase ROS as a downstream effect of JAK2 inhibition [32]. Collectively, these findings suggest that oxidative stress may serve as a key mediator of the cytotoxic and pro-apoptotic effects observed with **SL10** and **SL35** treatments, highlighting ROS induction as a potential therapeutic vulnerability in cervical cancer cells.

### 2.7. Structural Dynamics and Binding Free Energy Analysis of JAK2–SL Complexes

**Figure 7:**
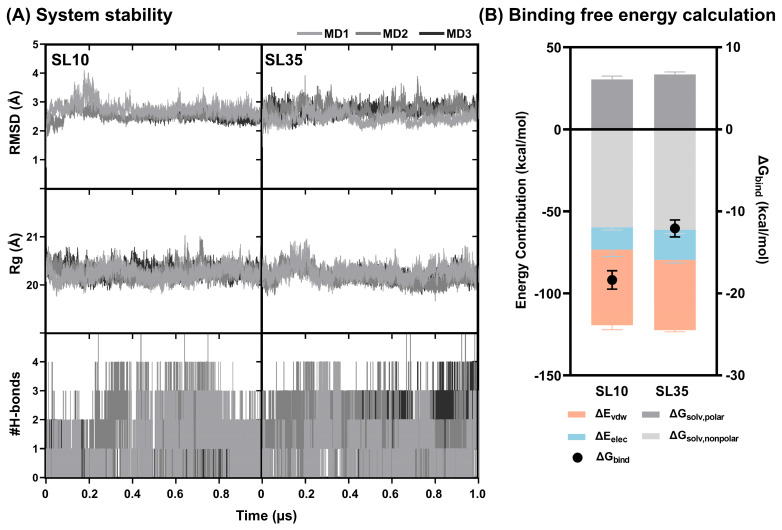
Structural stability and binding free energy of JAK2 in complex with **SL10** and **SL35** obtained from 1-µs MD simulations. (**A**) System stability assessed using root-mean-square deviation (RMSD), radius of gyration (Rg), and number of hydrogen bonds throughout triplicate simulations (MD1–MD3). (**B**) Binding free energy (ΔG_bind_) and energy decomposition calculated using the MM/GBSA method. Bars represent van der Waals energy (ΔE_vdw_), electrostatic energy (ΔE_elec_), polar solvation energy (ΔG_solv,polar_) and nonpolar solvation energy (ΔG_solv,non-polar_). The symbol ‘#’ indicates the number of hydrogen bonds.

The structural stability of the JAK2–ligand complexes was assessed via 1-μs MD simulations performed in triplicate (MD1–MD3). Root mean square deviation (RMSD), radius of gyration (R_g_) and the number of protein–ligand hydrogen bonds (#H-bonds) were analyzed to evaluate system equilibration and conformational stability (Figure 7A). Both complexes achieved equilibrium within ~300 ns, after which the trajectories remained stable throughout the remainder of the 1 μs simulations. To ensure that our analyses were based on the most reliable portion of the data, we focused on the 800–1000 ns of each trajectory. This approach minimizes the influence of initial equilibration fluctuations and ensures that the reported results reflect the stable phase of the simulations.

To further examine global compactness, the radius of gyration (R_g_) of the protein Cα atoms was monitored throughout the simulations. The average R_g_ remained stable at ~20.5 Å across all replicates, indicating maintenance of the protein’s overall structural integrity. Hydrogen bond analysis revealed that **SL10** formed more frequent and persistent hydrogen bonds with JAK2 than **SL35**, suggesting stronger and potentially more stable intermolecular interactions. This may contribute to **SL10**’s higher binding affinity and enhanced inhibitory potential. Collectively, these analyses confirm the stability and convergence of the simulated systems, supporting the validity of subsequent energetic and interaction analyses. Based on the equilibrated final 200 ns of each simulation, we further investigated (i) the binding affinity of **SL10** and **SL35** using MM/GBSA calculations, (ii) key interacting residues at the binding interface, (iii) protein–ligand hydrogen bonding patterns, and (iv) dynamic behavior of the protein–ligand complexes.

The MM/GBSA-calculated binding free energy and energy component breakdowns for **SL10** and **SL35** are shown in Figure 7B. The calculated van der Waals (ΔE_vdW_) values for **SL10** and **SL35** were −46.02 ± 2.77 kcal/mol and −42.83 ± 0.94 kcal/mol, respectively, which were significantly more negative than their corresponding electrostatic contributions (ΔE_ele_) of −13.66 ± 4.13 kcal/mol for **SL10** and −18.37 ± 1.84 kcal/mol for **SL35**. This indicates that van der Waals (vdW) interactions are the primary force driving complex formation, being approximately 2–3 times stronger than the electrostatic interactions in both compounds. The finding aligns with previous studies, which highlight the important role of hydrophobic interactions in protein-ligand binding, particularly in kinase interactions [53,87]. Considering solvation and entropy effects, **SL10** exhibited a substantially stronger binding free energy (ΔG_bind_) of −18.35 ± 1.12 kcal/mol, compared to −12.08 ± 1.63 kcal/mol for **SL35**. These computational findings are consistent with in vitro JAK2 kinase assay data, where **SL10** demonstrated a more potent inhibitory effect (IC_50_ = 12.70 ± 0.15 nM) than **SL35** (IC_50_ = 21.65 ± 0.21 nM). Altogether, these results suggest that enhancing vdW interaction energies may improve the overall ΔG_bind_, offering valuable insights for the rational design of more potent JAK2 inhibitors.

### 2.8. Interaction Profile of SL Derivatives as Potential JAK2 Inhibitors

**Figure 8:**
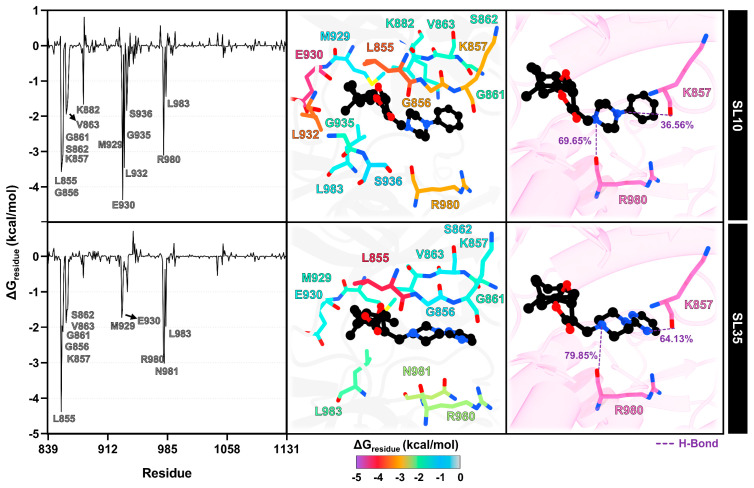
Binding interaction profiles and per-residue energy decomposition of JAK2 in complex with **SL10** and **SL35**. (**Left**): Per-residue free energy contributions (ΔG_residue_) calculated over the final 200 ns of MD simulations using MM/GBSA decomposition, (**Middle**): Key residues in the JAK2 binding site are color-coded based on their energetic contributions, (**Right**): Hydrogen bond interactions of **SL10** and **SL35** with the JAK2 active site.

The critical residues contributing to the binding of **SL10** and **SL35** were further characterized through per-residue free energy decomposition analysis (Figure 8). The majority of hot-spot residues were hydrophobic amino acids, which dominated the interaction profiles by contributing significantly to van der Waals and hydrophobic energies. Overall, 11–14 residues were identified as key contributors to binding, several of which were conserved between the two ligands. In particular, residues in the glycine-rich loop (V863, K857, G861, and G856) and the hinge region (M929 and E930) stabilized both **SL10** and **SL35**. These residues overlap substantially with the interaction pattern observed for the reference inhibitor ruxolitinib, which engages L855, V863, Y931, L932, and L983 [88], highlighting their importance in JAK2 inhibition. Residue-specific energy contributions further revealed distinct binding preferences for each ligand. For **SL10**, E930 emerged as the dominant contributor (ΔG_residue_ = −4.36 kcal/mol), followed by L855 (−3.57 kcal/mol), in agreement with the overall ΔG_bind_ values. In contrast, **SL35** binding was primarily stabilized by L855 (−4.09 kcal/mol), with van der Waals interactions providing the largest contribution. Notably, these residues have also been implicated in the binding of other JAK2 inhibitors, including imidazopyrrolopyridines [89] and aminopyrimidine derivatives [67], demonstrating their general relevance as pharmacophoric anchors within the JAK2 binding site.

In addition to hydrophobic contacts, hydrogen bonding interactions played a decisive role in complex stabilization. Using geometric criteria of ≤3.5 Å donor–acceptor distance and ≥120° bond angle, both **SL10** and **SL35** were found to form persistent hydrogen bonds with K857 in the glycine-rich loop and R980 in the hinge region (Figure 8). For **SL10**, hydrogen bond occupancies reached 36.56% with K857 and 69.65% with R980. **SL35** exhibited even stronger hydrogen bonding, with occupancies of 64.13% (K857) and 79.85% (R980). These findings highlight the dual contribution of hydrophobic contacts and directional hydrogen bonding in stabilizing JAK2–ligand complexes, with the hinge region and glycine-rich loop representing critical structural motifs for inhibitor design.

### 2.9. Conformational Dynamics of the JAK2 upon SL Binding

**Figure 9:**
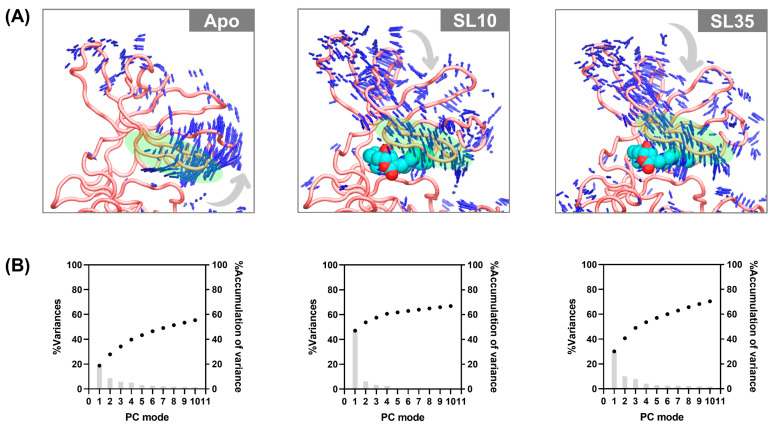
(**A**) PC1 Porcupine plot illustrating the apo form and the **SL10** and **SL35** complexes with JAK2, where the arrowhead indicates the direction of movement, and the arrow length reflects the magnitude of the motion. (**B**) PCA scree plot depicting the distribution of the principal components.

To explore the conformational changes in JAK2 upon binding with **SL10** and **SL35**, principal component analysis (PCA) was performed. As depicted in Figure 9, the first ten principal components (PCs) explained 55.33%, 66.86%, and 70.36% of the total variance in the apo JAK2, **SL10**/JAK2, and **SL35**/JAK2 complexes, respectively. Notably, PC1 accounted for a greater proportion of the variance compared to PC2 in both **SL10** and **SL35** complexes, suggesting that the major conformational alterations in the protein are captured by PC1. The structural changes in the protein were visualized through the porcupine plot of PC1 derived from the MD1 simulations for each complex. In the apo state, the glycine loop residues (highlighted in green) were displaced from the binding pocket, indicating an open conformation. Upon binding **SL10** and **SL35**, the protein adopted a closed conformation, stabilizing its active state.

## 3. Materials and Methods

### 3.1. Molecular Docking

Molecular docking is used to predict the binding orientation and affinity of small molecules within a protein’s active site, providing insights into potential inhibitory interactions [90]. Four crystal structures of JAK2 in complex with known inhibitors— tofacitinib (PDB ID: 3FUP [91]), ruxolitinib (PDB ID: 6VGL [92]), pacritinib (PDB ID: 8BPV [93]), and 3O4 (PDB ID: 4BBE [94])—were retrieved from the Protein Data Bank. Notably, 3O4 shares structural substituents with the **SL** compounds, while pacritinib also contains comparable moieties. Redocking validation, presented in Figure S16, revealed that each of the four ligand–protein complexes achieved RMSD values falling within the accepted 0–2 Å threshold, thereby confirming the robustness and accuracy of the docking approach [95,96]. Protonation states of JAK2 and all ligands were assigned using PDB2PQR [97] and MarvinSketch [98]. Ruxolitinib, a clinically approved ATP-competitive JAK inhibitor used as the reference drug, was obtained from the PubChem database [99]. Ligand geometries were optimized with Gaussian16 [100] at the Hartree–Fock (HF) level of theory employing the 6–31G(d) basis set, following established protocols [101,102]. For molecular docking, ligands were placed at the center of the JAK2 active site, and simulations were conducted using GOLD software, which applies a genetic algorithm (GA) for conformational sampling [103]. Docking was performed within a spherical region of 12 Å around the active site, generating 100 distinct poses for each ligand. Compounds with GOLD fitness scores exceeding that of ruxolitinib were prioritized for subsequent experimental validation.

### 3.2. Epoxidation of Alantolactone

General procedure: Sodium acetate (to pH 5–6) and a solution of alantolactone (10 mmol) in methylene chloride were added to a solution of freshly prepared peracetic acid obtained by cooling 5 mL of acetic anhydride, 1.1 mL of 30% hydrogen peroxide and 1 drop of sulfuric acid. The mixture was kept at room temperature while monitoring the degree of reaction by TLC, then poured into water, neutralized with a solution of NaHCO_3_ and extracted with chloroform (3 × 20 mL). To remove residual acetic anhydride, the organic layer was washed with water and after evaporation of the solvent; the residue was passed through a column of neutral aluminum oxide in benzene. As a result, (3aR,4S,4aR,5S,8aR,9aR)-5,8a-dimethyl-3-methylidene-4,4a-epoxy-3a,5,6,7,8,8a,9,9a-octahydronaphtho [2,3-b]furan-2(3H)-one (epoxyalantolactone) was obtained, yield 68%, the structure corresponds to that described in the literature [65,66].

### 3.3. Reaction of Epoxyalantolactone with Amines

General procedure: A solution of 1.1 mmol of amine in methanol (2 mL) was added with stirring to a solution of 1 mmol of epoxyalantolactone in methanol (5 mL) and left at room temperature, monitoring the degree of conversion of the initial alantolactone by TLC. If the product did not precipitate from the reaction mixture, the solvent was evaporated in vacuo and the residue was recrystallized from a suitable solvent (a mixture of benzene and hexane, commonly). The structural characterization for newly synthesized conjugates was confirmed by NMR spectroscopy (^1^H NMR and ^13^C NMR spectra shown in Supplementary Materials). For the derivatives already described (**SL14** and **SL33**), the chemical structures were confirmed based on literature data in [50] and [65], respectively. The spectra are provided in Figures S4–S15 in the Supplementary Materials. 

### 3.4. Cell Lines, Chemicals and Reagents

Human cervical carcinoma (HeLa, ATCC^®^ CCL-2™), mouse fibroblast (L929, ATCC^®^ CCL-1™), human hepatocellular carcinoma (HepG2, ATCC^®^ HB-8065™), and human neuroblastoma (SH-SY5Y, ATCC^®^ CRL-2266™) cell lines were obtained from the American Type Culture Collection (ATCC, Manassas, VA, USA). DMEM (Cat. No. 12100046) and DMEM/F-12 (Cat. No. 11320033) were purchased from Gibco™ ThermoFisher, Waltham, MA, USA. Fetal bovine serum (FBS) was obtained from Gibco™ ThermoFisher (Cat. No. A5256701). Penicillin-streptomycin was from Gibco™ ThermoFisher (Cat. No. 15140122). Trypsin-EDTA was purchased from Gibco™ (Cat. No. 25200056). Phosphate-buffered saline (PBS) was obtained from Gibco™, Thermo Fisher Scientific, Waltham, MA, USA (Cat. No. 10010023). The ADP-Glo™ Kinase Assay Kit was purchased from Promega, Madison, WI, USA (Cat. No. V9103). Poly (Glu:Tyr) peptide (4:1) was obtained from SignalChem, Richmond, BC, Canada (Cat. No. P61-58-500). JAK2 enzyme was purchased from Sigma-Aldrich, St. Louis, MO, USA. (Cat. No. SRP0171). Ruxolitinib was obtained from MedChemExpress, Monmouth Junction, NJ, USA (Cat. No. HY-50856). MTT was from Sigma-Aldrich, St. Louis, MO, USA. (Cat. No. M2128). Dimethyl sulfoxide (DMSO) was obtained from Sigma-Aldrich (Cat. No. D2650). 2′,7′-Dichlorodihydrofluorescein diacetate (DCFH-DA) was purchased from Sigma-Aldrich (Cat. No. D6883). Annexin V-FITC was from Invitrogen, Thermo Fisher Scientific, Carlsbad, CA, USA (Cat. No. A13199). HBSS was obtained from Sigma-Aldrich, St. Louis, MO, USA (Cat. No. H8264). Propidium iodide was purchased from BD Pharmingen, San Diego, CA, USA (Cat. No. 556463). Triton X-100 and sodium hydroxide (NaOH) were obtained from Sigma-Aldrich (Cat. No. T8787 and S8045, respectively). Hydrogen peroxide was obtained from Samchun Pure Chemical™, Pyeongtaek-si, Gyeonggi-do, Republic of Korea (CAS No. 7722-84-1). L-ascorbic acid was purchased from TCI Chemicals (Cat. No. A0011). Alantolactone was provided by the Laboratory of natural compounds of the IPAC RAS. Amines for modification of epoxyalantolactone were obtained from the compound library of InterBioScreen Ltd. (https://www.ibscreen.com).

### 3.5. JAK2 Kinase Assay

The JAK2 kinase activity was assessed using the ADP-Glo™ Kinase Assay (Promega Corporation) as previously described [53,101,102]. For each reaction, 2.5 ng/μL of JAK2 enzyme (SRP0171, Sigma-Aldrich) was combined with 1 μM of the test compounds or known drugs, 5 μM ATP, and 2 ng/μL of poly(glu·tyr) substrate in a reaction buffer containing 40 mM Tris-HCl (pH 7.5), 20 mM MgCl_2_, and 0.1 mg/mL BSA. The reaction mixture was incubated at room temperature for 1 h. After incubation, 5 μL of the ADP-Glo reagent was added to each reaction, followed by an additional 40 min incubation. Next, 10 μL of the kinase detection reagent was added, and the reactions were incubated at room temperature for 30 min to convert ADP to ATP. Luminescence, reflecting ATP levels, was measured using an EnSight Multimode Microplate Reader. The JAK2 kinase assay measures the ability of compounds to inhibit JAK2 enzymatic activity, typically by quantifying ATP consumption or ADP production using luminescent or colorimetric readouts. However, errors may arise from nonspecific inhibition, compound interference with the detection system, or variability in enzyme or substrate concentrations [104]. All assays were performed in triplicate, and the resulting data were presented as the relative inhibition (%) of inhibitors compared to the control without any inhibitor, as shown in Equation (1).

(1) $$\%\mathrm{Relative}\;\mathrm{inhibition}=\frac{\left[\left(\mathrm{positive}-\mathrm{negative}\right)-\left(\mathrm{sample}-\mathrm{negative}\right)\right]}{\left(\mathrm{positive}-\mathrm{negative}\right)}\times 100$$

### 3.6. Cell Culture

Human cervical adenocarcinoma (HeLa), hepatocellular carcinoma (HepG2) and mouse fibroblast (L929) cells were cultured in Dulbecco’s Modified Eagle’s Medium (DMEM) containing 10% fetal bovine serum (FBS), 100 U/mL penicillin G, and 100 μg/mL streptomycin. Human neuroblastoma (SH-SY5Y) cells were maintained in DMEM/F-12 medium with the same supplements. All cultures were incubated at 37 °C in a humidified atmosphere of 5% CO_2_.

### 3.7. Cytotoxicity Assay

Cytotoxicity assays evaluate the ability of compounds to reduce cell viability, typically by measuring metabolic activity [105]. Cells were seeded in 96-well plates at a density of 5 × 10^3^ cells per well in 100 µL of complete DMEM and incubated overnight at 37 °C in a humidified incubator with 5% CO_2_. The cells were then treated with 100 µL of **SL**s compounds or ruxolitinib at various concentrations. After 48 h of treatment, 100 µL of MTT solution (0.5 mg/mL in serum-free medium) was added to each well and incubated for an additional 3 h at 37 °C. The supernatant was then removed, and 100 µL of DMSO was added to dissolve the resulting formazan crystals. Absorbance was measured at 570 nm using an EnSight Multimode Microplate Reader (PerkinElmer, Waltham, MA, USA). IC_50_ values were calculated using nonlinear regression analysis in GraphPad Prism version 9.

### 3.8. Apoptosis Detection

Apoptosis detection assays are employed to evaluate programmed cell death, commonly using Annexin V/propidium iodide staining to distinguish early and late apoptotic populations. Possible limitations involve nonspecific binding of Annexin V/PI, difficulties in discriminating apoptotic from necrotic cells, and variations in flow cytometry calibration [106]. HeLa cells were seeded in 6-well plates at a density of 3 × 10^5^ cells per well in 2 mL of complete DMEM and allowed to adhere overnight. After 24 h, the cells were treated with **SL10** (8 µM), **SL35** (6 µM), or ruxolitinib (25 µM) for 48 h, corresponding to their respective IC_50_ values obtained from the cytotoxicity assay. Following treatment, cells were harvested by trypsinization and washed twice with 1 mL of cold phosphate-buffered saline (PBS) to remove non-adherent and dead cells. The cell pellets were resuspended in 100 µL of binding buffer and stained with 5 µL of Annexin V-FITC and 5 µL of propidium iodide (PI, 0.05 µg/mL). The staining was performed in the dark at room temperature for 15 min. Apoptotic cell death was quantified by flow cytometry, with early and late apoptotic populations distinguished based on Annexin V-FITC and PI fluorescence signals.

### 3.9. DCFH_2_-DA Assay

The DCFH_2_-DA assay is used to measure intracellular reactive oxygen species (ROS), where the non-fluorescent probe is converted into the fluorescent compound DCF upon oxidation [107]. HeLa cells were seeded in black 96-well plates at a density of 1 × 10^4^ cells per well and incubated overnight to allow cell attachment. After 24 h, the cells were incubated with 100 µL of 10 µM DCFH_2_-DA (Sigma-Aldrich) diluted in Hank’s Balanced Salt Solution (HBSS) at 37 °C for 30 min in the dark. Following dye loading, the cells were treated with either the IC_50_ concentrations of the test compounds, 200 µM hydrogen peroxide (H_2_O_2_; Samchun Pure Chemical™, Pyeongtaek-si, Gyeonggi-do, Republic of Korea), or 200 µM vitamin C (Acetic Acid; TCI Chemical, Tokyo, Japan #64-19-7) for 1 h. At the end of the treatment, cells were washed twice with cold phosphate-buffered saline (PBS), and 200 µL of 1% Triton X-100 in 0.3 M NaOH was added to lyse the cells. The fluorescence intensity, reflecting intracellular ROS levels, was measured using an EnSight Multimode Microplate Reader (PerkinElmer, Waltham, MA, USA) with excitation and emission wavelengths set at 485 nm and 535 nm, respectively. It should be noted that DCFH_2_-DA responds to a broad range of oxidative intermediates and does not distinguish among specific ROS and therefore reflects overall oxidative activity rather than species-specific ROS generation.

### 3.10. Molecular Dynamics Simulation

Molecular dynamics (MD) simulation is used to model the time-dependent behavior of biomolecular systems, providing insights into conformational flexibility, stability, and protein–ligand interactions under near-physiological conditions [108]. MD simulations on all ligand/JAK2 complexes were carried out with AMBER22 [109] under periodic boundary conditions, following methodologies outlined in previous research [88,101,102]. The electrostatic potential (ESP) charges for the optimized compounds were computed at the HF/6-31(d) level of theory and converted to restrained ESP (RESP) charges using the ANTECHAMBER module. The AMBER ff19SB force field was applied to the protein [110], while GAFF2 was utilized for the parameterization of the compounds [111]. The tLEaP module was used to add missing hydrogen atoms, and the system was energy-minimized using 1500 steps of both steepest descent (SD) and conjugate gradient (CG) methods to eliminate steric clashes. Counterions were added to neutralize the system, and the TIP3P water model was used to solvate the system within an octahedral box, ensuring at least a 10 Å buffer around the protein [112]. Following solvation, energy minimization was repeated on the explicit water molecules and counterions using the same SD and CG procedures. For molecular dynamics simulations, a 10 Å cutoff was set for short-range nonbonded interactions, while long-range electrostatic interactions were calculated using the Particle Mesh Ewald (PME) method [113]. The SHAKE algorithm was applied to constrain covalent bonds involving hydrogen atoms, ensuring stable integration. The system was gradually heated from 10 K to 310 K over 100 ps, followed by equilibration and a 1 µs production run conducted under periodic boundary conditions in the NPT ensemble using the AMBER 22 package [114]. The simulation trajectory were analyzed using the CPPTRAJ module [115] to assess the structural dynamics of the protein-ligand complex such as root mean square displacement (RMSD), radius of gyration (Rg), number of hydrogen bonding (H-bonds). Binding affinities were calculated using MM/GBSA [116]. The interactions between the potent **SL** inhibitors and JAK2 were examined using UCSF Chimera for comprehensive visualization of the binding conformations [117].

### 3.11. Pharmacokinetic and Toxicological Evaluation

Pharmacokinetic properties of the screened sesquiterpene lactone (**SL**) derivatives were predicted using SwissADME (http://www.swissadme.ch/) [118]. Toxicity profiles were evaluated using ProTox 3.0 (https://tox.charite.de/protox3/, accessed on 23 November 2025) [58], covering organ-specific toxicities (hepatotoxicity, nephrotoxicity, cardiotoxicity, neurotoxicity), mutagenicity, and cytotoxicity. Predicted LD_50_ values and toxicity classes were assigned based on Globally Harmonized System of Classification and Labelling of Chemicals (GHS) classification standards.

### 3.12. Statistical Analysis

The data are presented as the mean ± standard error of the mean (SEM) from three separate experiments. Statistical analyses were performed using one-way analysis of variance (ANOVA) with Tukey’s post hoc test. Statistical significance was defined as a *p*-value ≤ 0.05.

## 4. Conclusions

This study identified **SL10** and **SL35** as potent and selective sesquiterpene lactone derivatives targeting JAK2 through an integrated computational and experimental approach. Both compounds demonstrated strong cytotoxicity against HeLa cells by inducing apoptosis and elevating ROS levels, supporting their therapeutic potential in cervical cancer. Considering that only one cervical cancer cell line was examined, these findings should be interpreted as preliminary. Additional in vitro models will be required to confirm and extend their potential therapeutic significance. Binding free energy calculations underscored van der Waals interactions as the predominant stabilizing force, while per-residue energy decomposition revealed critical contributions from residues in the glycine-rich loop (K857, V863) and hinge region (E930, R980), consistent with the interaction profile of ruxolitinib. Stable hydrogen bonds with K857 and R980 further reinforced the inhibitory activity of both compounds. Collectively, these findings provide mechanistic insights into structure–activity relationships and highlight sesquiterpene lactone scaffolds as promising candidates for the development of targeted JAK2 inhibitors in cervical cancer therapy.

## Figures and Tables

**Table 1:** Drug-likeness parameters of the six screened sesquiterpene lactone derivatives based on Lipinski’s Rule of Five.

Compound	Drug-Likeness Parameters						
	MW ^a^	HBD ^b^	HBA ^c^	RB ^d^	PSA^e^	LogP ^f^	Lipinski ^g^
**SL8**	415.52	1	6	6	69.32	3.45	accept
**SL10**	410.55	0	4	3	45.31	3.38	accept
**SL14**	428.54	0	5	3	45.31	3.71	accept
**SL31**	424.58	0	4	3	45.31	3.72	accept
**SL33**	408.53	2	4	5	66.65	3.99	accept
**SL35**	412.53	0	6	3	71.09	2.33	accept

^a^ MW = molecular weight (Da): ≤500. ^b^ HBD = number of hydrogen-bond donors: ≤5. ^c^ HBA = number of hydrogen-bond acceptors: ≤10. ^d^ RB = number of rotatable bonds: ≤10. ^e^ PSA = topological polar surface area (Å^2^): ≤140. ^f^ LogP = log of octanol-to-water partition coefficient: ≤5. ^g^ Lipinski = Lipinski’s rule of five.

## Data Availability

The original contributions presented in this study are included in the article/Supplementary Materials. Further inquiries can be directed to the corresponding authors.

## Supplementary Materials

The following supporting information can be downloaded at: https://www.mdpi.com/article/10.3390/ijms27010329/s1.
