# Supplementary material for: Identification of Novel JAK2 Inhibitors from Amino Derivatives of Epoxyalantolactone: In Silico and In Vitro Studies
Source: Int J Mol Sci. 2025 Dec 28;27(1):329. doi: 10.3390/ijms27010329 (PMC12786078; doi:10.3390/ijms27010329)
Supplement: Supplementary file 1 [file ijms-27-00329-s001.zip › ijms-4040449-supplementary.pdf]

Supporting information

Identification of Novel JAK2 Inhibitors from Amino Derivatives of Epoxyalantolactone: In Silico and In Vitro Studies

Duangjai Todsaporn [1], Kamonpan Sanachai [2], Chanat Aonbangkhen [3], Rungtiva P. Poo-arporn [4], Victor Kartsev [5], Sergey Pukhov [6], Svetlana Afanasyeva [6], Athina Geronikaki [7,*] and Thanyada Rungrotmongkol [1,8,*]

[1] Center of Excellence in Structural and Computational Biology, Department of Biochemistry, Faculty of Science, Chulalongkorn University, Bangkok 10330, Thailand; fai.dt8@gmail.com

[2] Department of Biochemistry, Faculty of Science, Khon Kaen University, Khon Kaen 40002, Thailand; kamosa@kku.ac.th

[3] Center of Excellence in Natural Products Chemistry (CENP), Department of Chemistry, Faculty of Science, Chulalongkorn University, Bangkok 10330, Thailand; chanat.a@chula.ac.th

[4] Biological Engineering Program, Faculty of Engineering, King Mongkut's University of Technology Thonburi, Bangkok 10140, Thailand; rungtiva.pal@kmutt.ac.th

[5] InterBioScreen, Chernogolovka 142432, Russia; vkartsev@ibscreen.chg.ru

[6] Institute of Physiologically Active Compounds, Federal Research Center of Problems of Chemical Physics and Medicinal Chemistry, Russian Academy of Sciences, Chernogolovka 142432, Russia; serbg2007@yandex.ru (S.P.); svetlana.afanasyeva@yandex.ru (S.A.)

[7] Department of Pharmaceutical Chemistry, School of Pharmacy, Aristotle University of Thessaloniki, 54124 Thessaloniki, Greece

[8] Program in Bioinformatics and Computational Biology, Graduate School, Chulalongkorn University, Bangkok 10330, Thailand

* Correspondence: geronikaki@gmail.com or geronik@pharm.auth.gr (A.G.); thanyada.r@chula.ac.th or t.rungrotmongkol@gmail.com (T.R.)

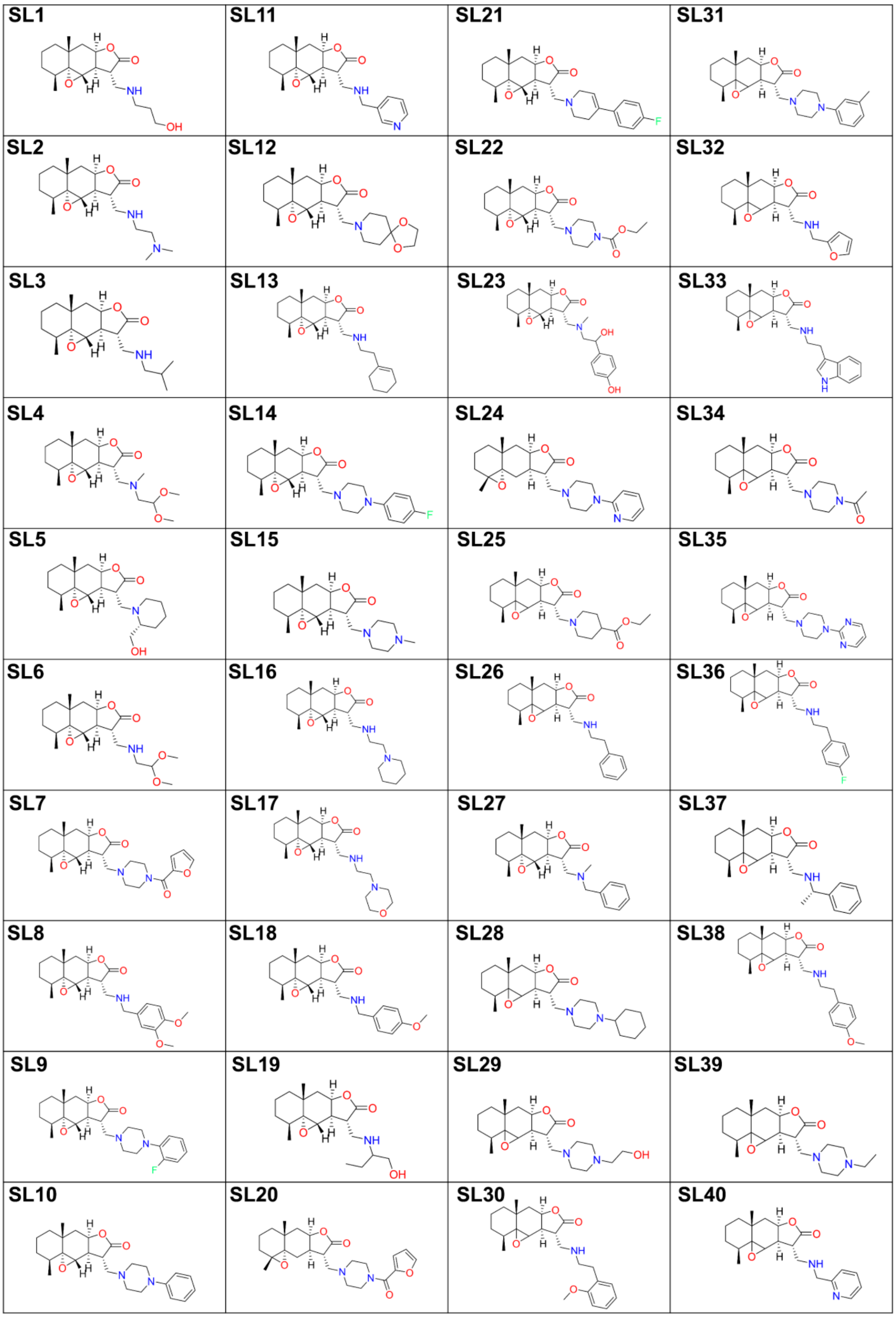

Figure S1. 2D structure of SL derivatives

Table S1. *In silico* toxicity prediction profiles of the selected sesquiterpene lactone (SL) derivatives. Each compound was evaluated for potential toxicity endpoints, including organ-specific toxicity (hepatotoxicity, nephrotoxicity, cardiotoxicity, neurotoxicity, and immunotoxicity), mutagenicity, and cytotoxicity. Both the predicted probabilities and binary classification (active/inactive) are reported. Acute toxicity was represented by predicted LD_{50} values (mg/kg) and toxicity class according to the Globally Harmonized System (GHS).

Compound	Target	Hepatotoxicity	Nephrotoxicity	Cardiotoxicity	Neurotoxicity	Immunotoxicity	Mutagenicity	Cytotoxicity	Predicted LD50 (mg/kg)	Class
SL8	Probability	0.83	0.54	0.59	0.6	0.99	0.61	0.58	1500	4
	Prediction	inactive	inactive	inactive	inactive	active	inactive	inactive		
SL10	Probability	0.83	0.54	0.87	0.7	0.9	0.62	0.71	700	4
	Prediction	inactive	inactive	inactive	active	active	inactive	inactive		
SL14	Probability	0.76	0.52	0.86	0.74	0.99	0.61	0.66	700	4
	Prediction	inactive	active	inactive	active	active	inactive	inactive		
SL31	Probability	0.82	0.51	0.87	0.67	0.95	0.64	0.71	1500	4
	Prediction	inactive	inactive	inactive	active	active	inactive	inactive		
SL33	Probability	0.8	0.61	0.68	0.57	0.99	0.56	0.7	800	4
	Prediction	inactive	inactive	inactive	active	active	inactive	inactive		
SL35	Probability	0.74	0.51	0.89	0.73	0.98	0.6	0.73	2000	4
	Prediction	inactive	inactive	inactive	active	active	inactive	inactive		

Table S2. Cross-docking results of screened compounds against JAK1, JAK2, JAK3 and TYK2

Compound	IC_{50} (μM)				
	HeLa	L929	HepG2	SH-SY5Y	HEK-293T
SL8	88.15 ± 5.66	92.63 ± 2.87	42.65 ± 2.14	59.65 ± 2.84	90.74 ± 1.61
SL10	8.47 ± 0.74	25.36 ± 2.35	19.68 ± 0.31	23.07 ± 0.85	14.54 ± 0.74
SL14	13.18 ± 1.07	52.75 ± 1.92	27.52 ± 0.73	35.75 ± 2.13	50.85 ± 3.30
SL31	13.71 ± 1.04	62.80 ± 3.39	19.62 ± 0.05	24.20 ± 1.69	24.47 ± 1.12
SLL33	33.78 ± 2.78	40.53 ± 2.22	45.28 ± 0.41	58.60 ± 0.71	29.32 ± 0.96
SL35	6.07 ± 0.11	23.05 ± 0.50	13.32 ± 1.12	19.25 ± 1.40	14.64 ± 1.05
Ruxolitinib	25.02 ± 1.25	80.36 ± 2.33	34.55 ± 2.56	68.63 ± 0.19	89.73 ± 1.98

Table S3. Comparison of cytotoxic effects of sesquiterpene lactone derivatives and ruxolitinib across cervical cancer (HeLa), hepatocellular carcinoma (HepG2), neuroblastoma (SH-SY5Y), embryonic kidney epithelial (HEK-293T), and mouse fibroblast (L929) cell lines. IC_{50} values are presented as mean ± SEM from triplicate experiments.

Compound	GOLD fitness score			
	JAK1 (PDB ID:6N7D)	JAK2 (PDB ID:3FUP)	JAK3 (PDB ID:5TOZ)	TYK2 (PDB ID:4GJ2)
**Ruxolitinib**	58.58	60.48	52.46	53.26
**SL8**	55.11	61.73	51.73	54.57
**SL10**	56.66	61.09	47.68	55.25
**SL14**	65.22	64.83	56.14	56.55
**SL31**	65.31	64.43	50.21	54.93
**SL33**	63.8	63.09	57.83	53.44
**SL35**	59.9	61.03	55.21	53.32

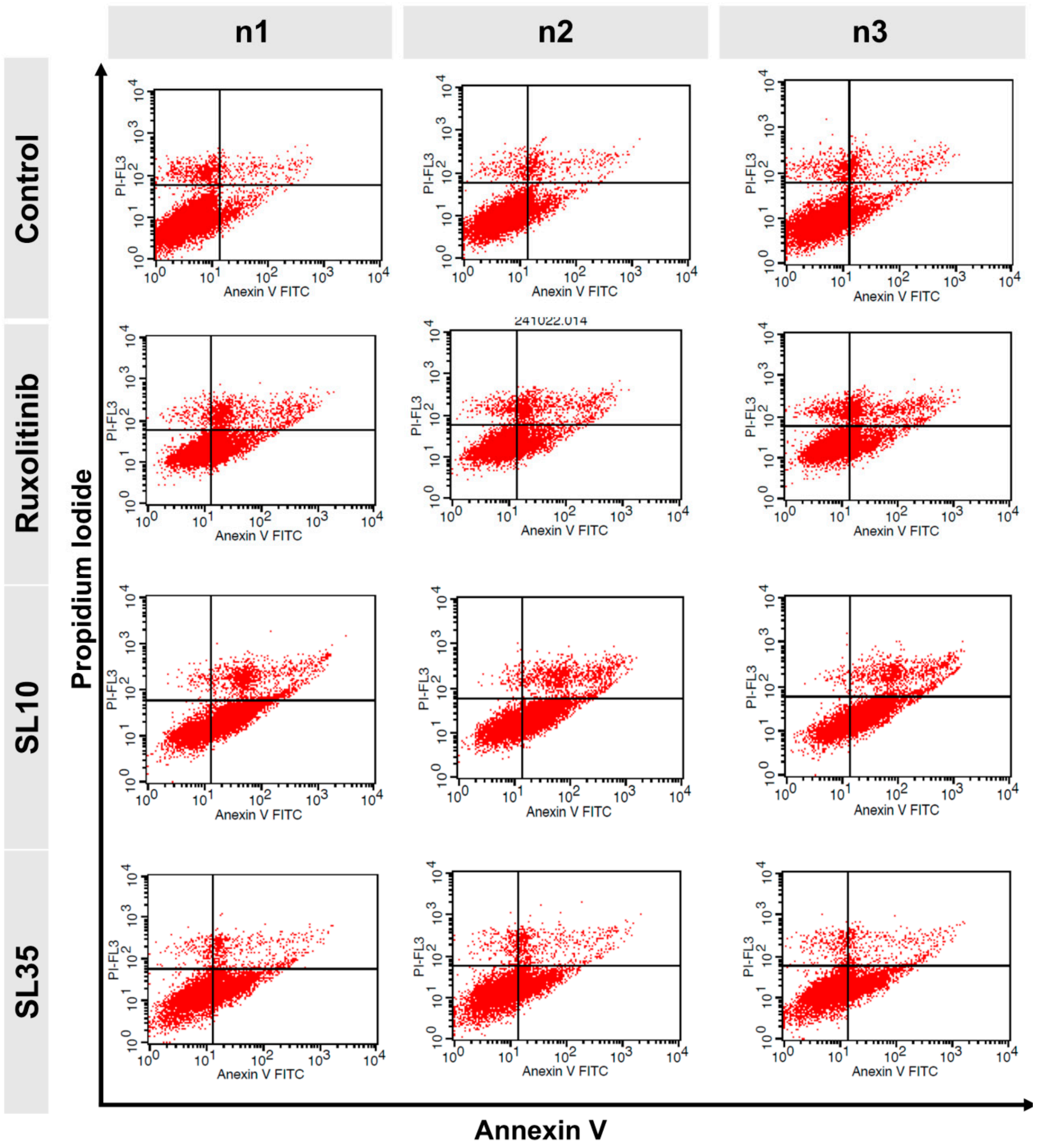

Figure S2. Flow cytometry analysis of apoptosis induction in HeLa cells treated with ruxolitinib, SL10, or SL35 using Annexin V–FITC/propidium iodide (PI) dual staining. Representative dot plots from three independent experiments (n1–n3) are shown. The lower left quadrant represents viable cells (Annexin V^{-}/PI^{-}), the lower right quadrant represents early apoptotic cells (Annexin V^{+}/PI^{-}), the upper right quadrant represents late apoptotic/necrotic cells (Annexin V^{+}/PI^{+}), and the upper left quadrant represents necrotic cells (Annexin V^{-}/PI^{+}).

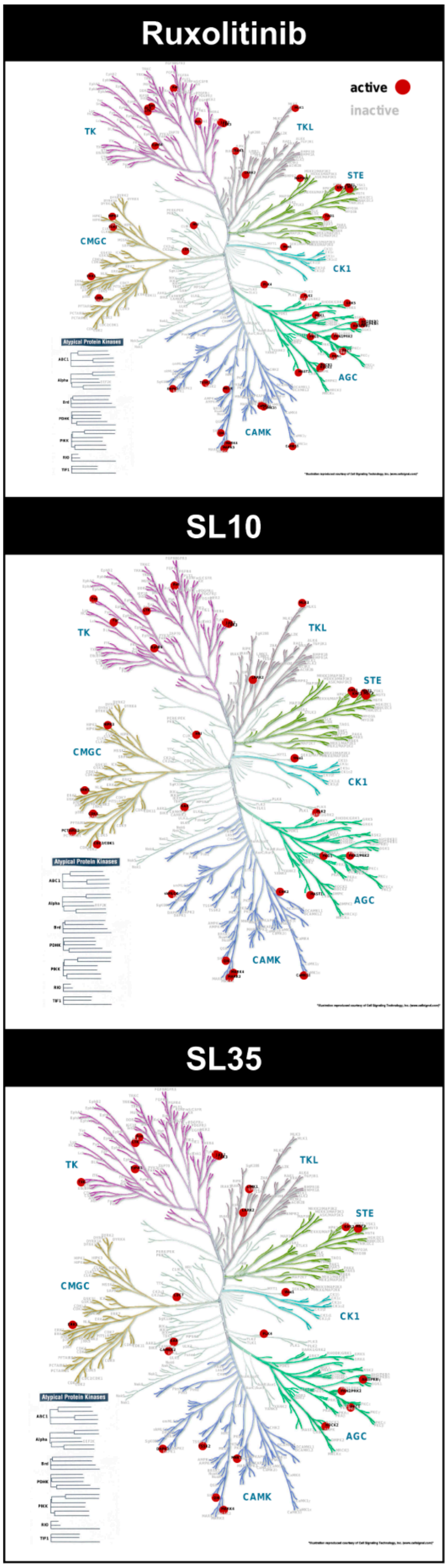

Figure S3. Prediction of the kinase selectivity of Ruxolitinib, SL10, and SL35

Table S4. Predicted binding probabilities of SL10, SL35, and ruxolitinib against a panel of human kinases from KinomeX platform.

No.	Protein	SL10	SL35	Ruxolitinib
1	hAAK1(Q2M2I8)	0.20	0.36	1.00
2	hABL1(P00519)	0.03	0.47	1.00
3	hABL2(P42684)	0.00	0.27	1.00
4	hACVR2A(P27037)	0.01	0.34	1.00
5	hACVR2B(Q13705)	0.00	0.34	1.00
6	hADRBK1(P25098)	0.05	0.29	1.00
7	hAKT1(P31749)	0.10	0.56	1.00
8	hAKT2(P31751)	0.03	0.60	0.99
9	hAKT3(Q9Y243)	0.01	0.77	0.99
10	hALK(Q9UM73)	0.30	0.44	0.99
11	hALK1(P37023)	0.00	0.34	0.99
12	hALK2(Q04771)	0.13	0.66	0.99
13	hALK4(P36896)	0.16	0.58	0.98
14	hALS2CR7(Q96Q40)	0.05	0.42	0.98
15	hANKK1(Q8NFD2)	0.22	0.36	0.98
16	hAURa(O14965)	0.05	0.30	0.97
17	hAURb(Q96GD4)	0.32	0.35	0.97
18	hAURc(Q9UQB9)	0.69	0.39	0.96
19	hAXL(P30530)	0.41	0.44	0.95
20	hBLK(P51451)	0.25	0.31	0.94
21	hBMP2K(Q9NSY1)	0.21	0.37	0.94
22	hBMPR1A(P36894)	0.01	0.33	0.93
23	hBMPR1B(O00238)	0.41	0.56	0.93
24	hBMPR2(Q13873)	0.01	0.19	0.92
25	hBMX(P51813)	0.01	0.48	0.91
26	hBRAF(P15056)	0.01	0.16	0.90
27	hBRSK1(Q8TDC3)	0.01	0.44	0.90
28	hBTK(Q06187)	0.00	0.36	0.89
29	hCAMK1(Q14012)	0.00	0.23	0.89
30	hCAMK1D(Q8IU85)	0.99	0.68	0.88
31	hCAMK1G(Q96NX5)	0.18	0.34	0.88
32	hCAMK2A(Q9UQM7)	0.08	0.29	0.87
33	hCAMK2B(Q13554)	0.00	0.25	0.87
34	hCAMK2D(Q13557)	0.04	0.38	0.87
35	hCAMK2G(Q13555)	0.02	0.21	0.86
36	hCAMK4(Q16566)	0.01	0.10	0.86
37	hCAMKK1(Q8N5S9)	0.58	0.62	0.83
38	hCAMKK2(Q96RR4)	0.40	0.74	0.82
39	hCDC2L6(Q9BWU1)	0.00	0.13	0.82

No.	Protein	SL10	SL35	Ruxolitinib
40	hCDC42BPA(Q5VT25)	0.01	0.43	0.81
41	hCDC42BPB(Q9Y5S2)	0.05	0.38	0.80
42	hCDC42BPG(Q6DT37)	0.00	0.23	0.78
43	hCDC7(O00311)	0.12	0.79	0.76
44	hCDK1(P06493)	0.76	0.38	0.75
45	hCDK2(P24941)	0.31	0.38	0.75
46	hCDK3(Q00526)	0.40	0.53	0.71
47	hCDK4(P11802)	0.88	0.36	0.70
48	hCDK5(Q00535)	0.00	0.26	0.70
49	hCDK6(Q00534)	0.04	0.41	0.68
50	hCDK7(P50613)	0.13	0.15	0.68
51	hCDK8(P49336)	0.01	0.13	0.66
52	hCDK9(P50750)	0.10	0.23	0.66
53	hCDKL2(Q92772)	0.49	0.59	0.65
54	hCDKL3(Q8IVW4)	0.46	0.59	0.65
55	hCDKL5(O76039)	0.34	0.56	0.64
56	hCHUK(O15111)	0.02	0.43	0.64
57	hCIT(O14578)	0.03	0.31	0.64
58	hCLK1(P49759)	0.39	0.44	0.64
59	hCLK2(P49760)	0.02	0.29	0.64
60	hCLK3(P49761)	0.00	0.09	0.61
61	hCLK4(Q9HAZ1)	0.41	0.46	0.61
62	hCSF1R(P07333)	0.12	0.17	0.61
63	hCSK(P41240)	0.00	0.29	0.60
64	hCSNK1A1(P48729)	0.00	0.31	0.59
65	hCSNK1A1L(Q8N752)	0.20	0.36	0.59
66	hCSNK1D(P48730)	0.00	0.14	0.58
67	hCSNK1E(P49674)	0.25	0.48	0.58
68	hCSNK1G1(Q9HCP0)	0.04	0.32	0.57
69	hCSNK1G2(P78368)	0.01	0.31	0.56
70	hCSNK1G3(Q9Y6M4)	0.00	0.02	0.55
71	hCSNK2A1(P68400)	0.00	0.37	0.55
72	hCSNK2A2(P19784)	0.00	0.33	0.55
73	hChk1(O14757)	0.11	0.57	0.53
74	hChk2(O96017)	0.79	0.58	0.53
75	hDAPK1(P53355)	0.07	0.25	0.51
76	hDAPK2(Q9UIK4)	0.02	0.22	0.51
77	hDAPK3(O43293)	0.19	0.77	0.51
78	hDCAMKL1(O15075)	0.00	0.12	0.51
79	hDCAMKL2(Q8N568)	0.07	0.30	0.51
80	hDCAMKL3(Q9C098)	0.02	0.24	0.50
81	hDDR1(Q08345)	0.12	0.60	0.49

No.	Protein	SL10	SL35	Ruxolitinib
82	hDMPK(Q09013)	0.01	0.13	0.48
83	hDYRK1A(Q13627)	0.04	0.19	0.48
84	hDYRK1B(Q9Y463)	0.25	0.39	0.47
85	hDYRK2(Q92630)	0.23	0.25	0.47
86	hDYRK3(O43781)	0.02	0.36	0.47
87	hDYRK4(Q9NR20)	0.00	0.24	0.47
88	hEEF2K(O00418)	0.00	0.07	0.46
89	hEGFR(P00533)	0.00	0.15	0.45
90	hEIF2AK2(P19525)	0.23	0.38	0.44
91	hEPHA1(P21709)	0.63	0.74	0.42
92	hEPHA2(P29317)	0.00	0.08	0.42
93	hEPHA3(P29320)	0.03	0.68	0.40
94	hEPHA4(P54764)	0.03	0.45	0.38
95	hEPHA5(P54756)	0.00	0.62	0.38
96	hEPHA7(Q15375)	0.62	0.44	0.37
97	hEPHA8(P29322)	0.04	0.40	0.37
98	hEPHB1(P54762)	0.18	0.39	0.37
99	hEPHB2(P29323)	0.00	0.19	0.36
100	hEPHB3(P54753)	0.00	0.45	0.36
101	hEPHB4(P54760)	0.02	0.36	0.36
102	hEPHB6(O15197)	0.77	0.59	0.34
103	hERK1(P27361)	0.53	0.31	0.34
104	hERK2(P28482)	0.00	0.10	0.34
105	hERK3(Q16659)	0.08	0.34	0.33
106	hERK5(Q13164)	0.87	0.70	0.33
107	hERK7(Q8TD08)	0.61	0.30	0.31
108	hFAK1(Q05397)	0.01	0.26	0.29
109	hFAK2(Q14289)	0.25	0.32	0.28
110	hFER(P16591)	0.44	0.48	0.28
111	hFES(P07332)	0.01	0.43	0.27
112	hFGFR1(P11362)	0.03	0.06	0.26
113	hFGFR2(P21802)	0.50	0.21	0.25
114	hFGFR3(P22607)	0.05	0.18	0.25
115	hFGFR4(P22455)	0.00	0.07	0.24
116	hFGR(P09769)	0.79	0.42	0.22
117	hFLT3(P36888)	0.02	0.12	0.22
118	hFRK(P42685)	0.41	0.49	0.22
119	hFYN(P06241)	0.04	0.14	0.21
120	hGAK(O14976)	0.77	0.84	0.20
121	hGRK1(Q15835)	0.57	0.54	0.20
122	hGRK4(P32298)	0.58	0.52	0.20
123	hGRK5(P34947)	0.69	0.56	0.19

No.	Protein	SL10	SL35	Ruxolitinib
124	hGRK7(Q8WTQ7)	0.53	0.51	0.19
125	hGSK3a(P49840)	0.04	0.09	0.19
126	hGSK3b(P49841)	0.09	0.12	0.18
127	hHCK(P08631)	0.40	0.57	0.18
128	hHER2(P04626)	0.00	0.12	0.18
129	hHER4(Q15303)	0.00	0.10	0.18
130	hHIPK1(Q86Z02)	0.03	0.46	0.18
131	hHIPK2(Q9H2X6)	0.02	0.32	0.18
132	hHIPK3(Q9H422)	0.76	0.60	0.18
133	hHIPK4(Q8NE63)	0.01	0.10	0.18
134	hHRI(Q9BQI3)	0.99	0.52	0.18
135	hICK(Q9UPZ9)	0.69	0.57	0.17
136	hIGF1R(P08069)	0.01	0.64	0.17
137	hIKBKB(O14920)	0.24	0.45	0.16
138	hIKBKE(Q14164)	0.00	0.17	0.16
139	hINSR(P06213)	0.00	0.64	0.16
140	hINSRR(P14616)	0.03	0.28	0.15
141	hIRAK1(P51617)	0.08	0.69	0.15
142	hIRAK3(Q9Y616)	0.03	0.25	0.15
143	hIRAK4(Q9NWZ3)	0.00	0.53	0.15
144	hITK(Q08881)	0.23	0.26	0.15
145	hJAK2(O60674)	0.99	0.84	0.15
146	hJAK3(P52333)	1.00	0.83	0.15
147	hJNK1(P45983)	0.09	0.15	0.15
148	hJNK2(P45984)	0.00	0.05	0.15
149	hJNK3(P53779)	0.42	0.68	0.15
150	hKIT(P10721)	0.02	0.14	0.15
151	hLATS1(O95835)	0.26	0.38	0.15
152	hLCK(P06239)	0.02	0.22	0.15
153	hLIMK1(P53667)	0.37	0.73	0.15
154	hLIMK2(P53671)	0.15	0.39	0.14
155	hLRRK2(Q5S007)	0.95	0.84	0.14
156	hLTK(P29376)	0.99	0.86	0.14
157	hLYN(P07948)	0.01	0.08	0.14
158	hMAP3K10(Q02779)	0.23	0.64	0.14
159	hMAP3K11(Q16584)	0.71	0.37	0.13
160	hMAP3K13(O43283)	0.61	0.54	0.13
161	hMAP3K14(Q99558)	0.01	0.20	0.13
162	hMAP3K15(Q6ZN16)	0.12	0.50	0.13
163	hMAP3K2(Q9Y2U5)	0.55	0.53	0.12
164	hMAP3K3(Q99759)	0.23	0.45	0.12
165	hMAP3K4(Q9Y6R4)	0.00	0.33	0.12

No.	Protein	SL10	SL35	Ruxolitinib
166	hMAP3K5(Q99683)	0.00	0.03	0.12
167	hMAP3K6(O95382)	0.34	0.54	0.11
168	hMAP3K9(P80192)	0.35	0.66	0.11
169	hMAP4K1(Q92918)	0.33	0.48	0.11
170	hMAP4K2(Q12851)	0.14	0.61	0.11
171	hMAP4K3(Q8IVH8)	1.00	0.79	0.11
172	hMAP4K4(O95819)	0.14	0.51	0.11
173	hMAP4K5(Q9Y4K4)	0.91	0.43	0.10
174	hMAPKAPK3(Q16644)	0.00	0.09	0.10
175	hMAPKAPK5(Q8IW41)	0.00	0.06	0.10
176	hMARK1(Q9P0L2)	0.03	0.33	0.10
177	hMARK2(Q7KZI7)	0.54	0.31	0.10
178	hMARK3(P27448)	0.97	0.61	0.09
179	hMARK4(Q96L34)	0.85	0.85	0.09
180	hMAST1(Q9Y2H9)	0.84	0.59	0.09
181	hMATK(P42679)	0.00	0.19	0.09
182	hMEK1(Q02750)	0.18	0.59	0.09
183	hMEK2(P36507)	0.05	0.19	0.08
184	hMELK(Q14680)	0.49	0.44	0.08
185	hMET(P08581)	0.00	0.60	0.08
186	hMINK1(Q8N4C8)	0.41	0.46	0.08
187	hMK2(P49137)	0.00	0.14	0.08
188	hMKK3(P46734)	0.06	0.40	0.08
189	hMKK4(P45985)	0.02	0.32	0.07
190	hMKK6(P52564)	0.01	0.28	0.07
191	hMKK7(O14733)	0.06	0.28	0.07
192	hMKNK1(Q9BUB5)	0.03	0.77	0.06
193	hMKNK2(Q9HBH9)	0.03	0.58	0.06
194	hMPS1(P33981)	0.05	0.26	0.06
195	hMSK1(O75582)	0.03	0.46	0.06
196	hMSK2(O75676)	0.68	0.38	0.06
197	hMSSK1(Q9UPE1)	0.27	0.45	0.05
198	hMST1R(Q04912)	0.48	0.66	0.05
199	hMST4(Q9P289)	0.02	0.18	0.05
200	hMUSK(O15146)	0.01	0.14	0.05
201	hMYLK(Q15746)	0.04	0.32	0.04
202	hMYLK2(Q9H1R3)	0.87	0.51	0.04
203	hMYO3A(Q8NEV4)	0.08	0.42	0.04
204	hMYO3B(Q8WXR4)	0.05	0.39	0.04
205	hNEK1(Q96PY6)	0.00	0.20	0.04
206	hNEK2(P51955)	0.00	0.32	0.04
207	hNEK4(P51957)	0.00	0.37	0.04

No.	Protein	SL10	SL35	Ruxolitinib
208	hNEK5(Q6P3R8)	0.04	0.40	0.04
209	hNEK6(Q9HC98)	0.00	0.36	0.04
210	hNEK7(Q8TDX7)	0.02	0.33	0.04
211	hNIM1(Q8IY84)	0.03	0.23	0.04
212	hNLK(Q9UBE8)	0.68	0.49	0.04
213	hNTRK1(P04629)	0.08	0.11	0.03
214	hNTRK2(Q16620)	0.02	0.06	0.03
215	hNTRK3(Q16288)	0.10	0.14	0.03
216	hNUAK1(O60285)	0.01	0.13	0.03
217	hNUAK2(Q9H093)	0.21	0.35	0.03
218	hOXSR1(O95747)	0.41	0.49	0.03
219	hPAK1(Q13153)	0.00	0.22	0.03
220	hPAK2(Q13177)	0.00	0.36	0.03
221	hPAK3(O75914)	0.01	0.25	0.03
222	hPAK4(O96013)	0.00	0.31	0.03
223	hPAK6(Q9NQU5)	0.09	0.40	0.03
224	hPAK7(Q9P286)	0.27	0.39	0.03
225	hPASK(Q96RG2)	0.67	0.57	0.02
226	hPBK(Q96KB5)	0.00	0.41	0.02
227	hPCTK1(Q00536)	0.29	0.35	0.02
228	hPCTK2(Q00537)	0.75	0.50	0.02
229	hPDGFRa(P16234)	0.00	0.22	0.02
230	hPDGFRb(P09619)	0.05	0.16	0.02
231	hPDPK1(O15530)	0.16	0.55	0.02
232	hPEK(Q9NZJ5)	0.33	0.52	0.02
233	hPHKG1(Q16816)	0.08	0.27	0.02
234	hPHKG2(P15735)	0.00	0.45	0.02
235	hPIM1(P11309)	0.07	0.56	0.02
236	hPIM2(Q9P1W9)	0.00	0.31	0.02
237	hPIM3(Q86V86)	0.00	0.47	0.02
238	hPITSLREa(P21127)	0.06	0.33	0.02
239	hPITSLREb(Q9UQ88)	0.02	0.16	0.02
240	hPKAa(P17612)	0.12	0.62	0.02
241	hPKAb(P22694)	0.00	0.46	0.01
242	hPKAg(P22612)	0.00	0.47	0.01
243	hPKCa(P17252)	0.02	0.05	0.01
244	hPKCb1(P05771)	0.01	0.24	0.01
245	hPKCd(Q05655)	0.46	0.86	0.01
246	hPKCe(Q02156)	0.24	0.40	0.01
247	hPKCg(P05129)	0.05	0.32	0.01
248	hPKCi(P41743)	0.00	0.07	0.01
249	hPKCt(Q04759)	0.37	0.46	0.01

No.	Protein	SL10	SL35	Ruxolitinib
250	hPKCz(Q05513)	0.00	0.06	0.01
251	hPKD1(Q15139)	0.02	0.21	0.01
252	hPKD2(Q9BZL6)	0.00	0.43	0.01
253	hPKD3(O94806)	0.00	0.34	0.01
254	hPKMYT1(Q99640)	0.00	0.40	0.01
255	hPKN1(Q16512)	0.06	0.26	0.01
256	hPKN2(Q16513)	0.86	0.71	0.01
257	hPLK1(P53350)	0.02	0.16	0.01
258	hPLK2(Q9NYY3)	0.80	0.66	0.01
259	hPLK3(Q9H4B4)	0.00	0.11	0.01
260	hPLK4(O00444)	0.67	0.76	0.01
261	hPRKAA1(Q13131)	0.59	0.52	0.01
262	hPRKAA2(P54646)	0.06	0.30	0.01
263	hPRKG1(Q13976)	0.71	0.68	0.01
264	hPRKG2(Q13237)	0.00	0.48	0.01
265	hPRKX(P51817)	0.16	0.33	0.01
266	hPTK6(Q13882)	0.00	0.09	0.01
267	hQSK(Q9Y2K2)	0.31	0.52	0.01
268	hRAF1(P04049)	0.51	0.45	0.01
269	hRET(P07949)	0.74	0.64	0.01
270	hRIPK1(Q13546)	0.07	0.47	0.01
271	hRIPK2(O43353)	0.01	0.62	0.01
272	hROCK1(Q13464)	0.22	0.67	0.01
273	hROCK2(O75116)	0.05	0.70	0.01
274	hROS1(P08922)	0.20	0.72	0.01
275	hRPS6KB1(P23443)	0.65	0.41	0.01
276	hRPS6KB2(Q9UBS0)	0.13	0.12	0.01
277	hRSK2(P51812)	0.55	0.69	0.01
278	hSGK(O00141)	0.00	0.21	0.01
279	hSLK(Q9H2G2)	0.23	0.40	0.01
280	hSNF1LK(P57059)	0.48	0.66	0.01
281	hSNF1LK2(Q9H0K1)	0.93	0.84	0.00
282	hSRC(P12931)	0.15	0.64	0.00
283	hSRMS(Q9H3Y6)	0.00	0.22	0.00
284	hSRPK1(Q96SB4)	0.01	0.16	0.00
285	hSTK10(O94804)	0.01	0.30	0.00
286	hSTK11(Q15831)	0.28	0.38	0.00
287	hSTK16(O75716)	0.21	0.36	0.00
288	hSTK17A(Q9UEE5)	0.50	0.53	0.00
289	hSTK17B(O94768)	0.67	0.42	0.00
290	hSTK24(Q9Y6E0)	0.25	0.37	0.00
291	hSTK25(O00506)	0.27	0.39	0.00

No.	Protein	SL10	SL35	Ruxolitinib
292	hSTK3(Q13188)	0.99	0.76	0.00
293	hSTK32B(Q9NY57)	0.00	0.26	0.00
294	hSTK32C(Q86UX6)	0.00	0.26	0.00
295	hSTK33(Q9BYT3)	0.01	0.34	0.00
296	hSTK35(Q8TDR2)	0.32	0.55	0.00
297	hSTK36(Q9NRP7)	0.12	0.51	0.00
298	hSTK38(Q15208)	0.11	0.36	0.00
299	hSTK38L(Q9Y2H1)	0.32	0.40	0.00
300	hSTK39(Q9UEW8)	0.68	0.56	0.00
301	hSTK4(Q13043)	0.99	0.63	0.00
302	hSYK(P43405)	0.00	0.54	0.00
303	hSgK085(Q86YV6)	0.26	0.38	0.00
304	hSgK110(P0C264)	0.35	0.57	0.00
305	hTAOK1(Q7L7X3)	0.05	0.36	0.00
306	hTAOK3(Q9H2K8)	0.44	0.41	0.00
307	hTBK1(Q9UHD2)	0.00	0.18	0.00
308	hTESK1(Q15569)	0.22	0.57	0.00
309	hTGFbR1(P36897)	0.01	0.49	0.00
310	hTIE1(P35590)	0.02	0.17	0.00
311	hTIE2(Q02763)	0.03	0.07	0.00
312	hTLK1(Q9UKI8)	0.29	0.37	0.00
313	hTLK2(Q86UE8)	0.14	0.35	0.00
314	hTNIK(Q9UKE5)	0.22	0.36	0.00
315	hTNK1(Q13470)	0.20	0.36	0.00
316	hTNK2(Q07912)	0.03	0.69	0.00
317	hTNNI3K(Q59H18)	0.03	0.38	0.00
318	hTSSK1(Q9BXA7)	0.06	0.34	0.00
319	hTSSK2(Q96PF2)	0.00	0.79	0.00
320	hTXK(P42681)	0.11	0.87	0.00
321	hTYK2(P29597)	0.05	0.27	0.00
322	hTYRO3(Q06418)	0.00	0.59	0.00
323	hULK1(O75385)	0.68	0.57	0.00
324	hULK2(Q8IYT8)	0.67	0.56	0.00
325	hULK3(Q6PHR2)	0.67	0.57	0.00
326	hVEGFR1(P17948)	0.20	0.15	0.00
327	hVEGFR2(P35968)	0.37	0.17	0.00
328	hVEGFR3(P35916)	0.31	0.13	0.00
329	hWEE1(P30291)	0.92	0.78	0.00
330	hYES1(P07947)	0.84	0.68	0.00
331	hYSK4(Q56UN5)	0.13	0.49	0.00
332	hZAK(Q9NYL2)	0.00	0.06	0.00
333	hZAP70(P43403)	0.00	0.22	0.00

No.	Protein	SL10	SL35	Ruxolitinib
334	hp38a(Q16539)	0.00	0.08	0.00
335	hp38d(O15264)	0.00	0.04	0.00
336	hp38g(P53778)	0.00	0.07	0.00

^{1}H and ^{13}C NMR spectra (δ, ppm, J, Hz) were recorded on a Quantum-I Plus 400 spec-trometer in CDCl3 with Me4Si as an internal standard; the lower indices alpha and beta denote nonequivalent protons at one carbon atom. The numbering of atoms in the formula is given in accordance with the traditionally established nomenclature.

SL8

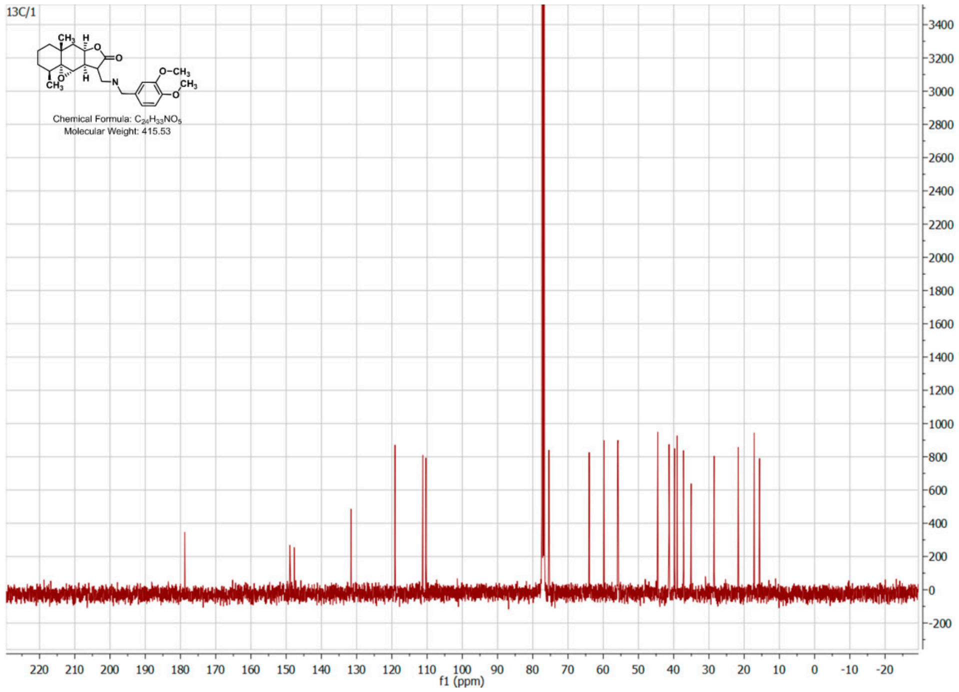

Figure S4. ^{13}C NMR spectra of SL8

SL10

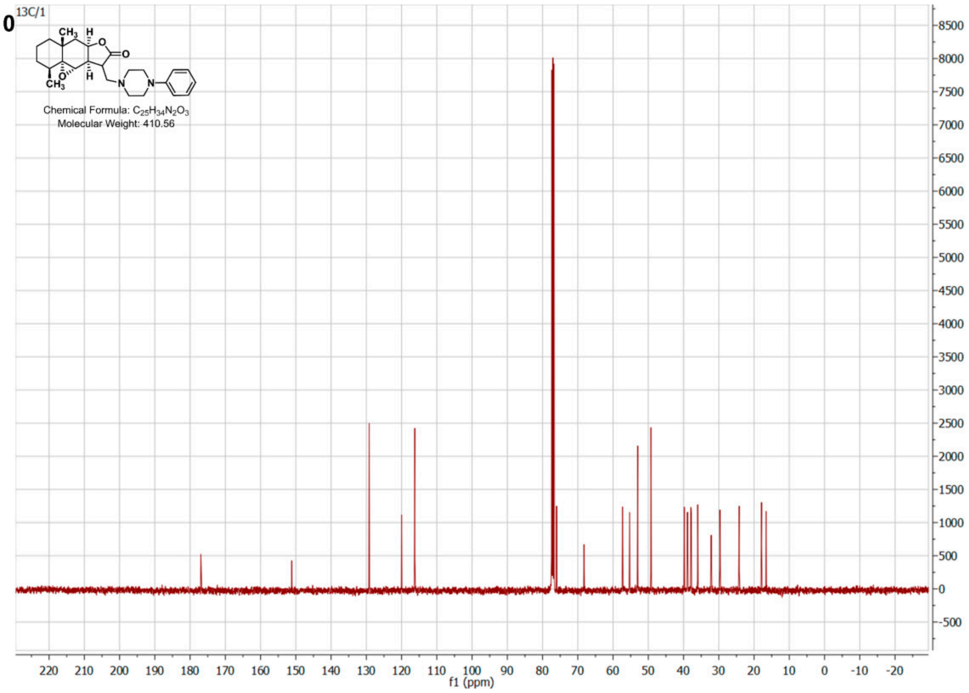

Figure S5. ^{13}C NMR spectra of SL10

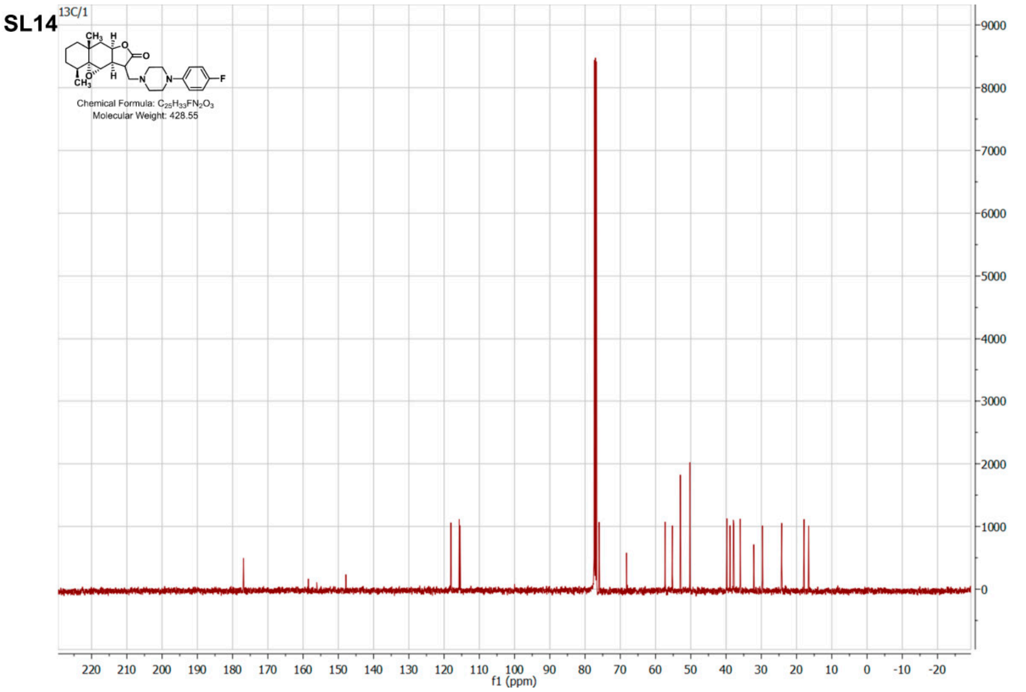

Figure S6. 13C NMR spectra of SL14

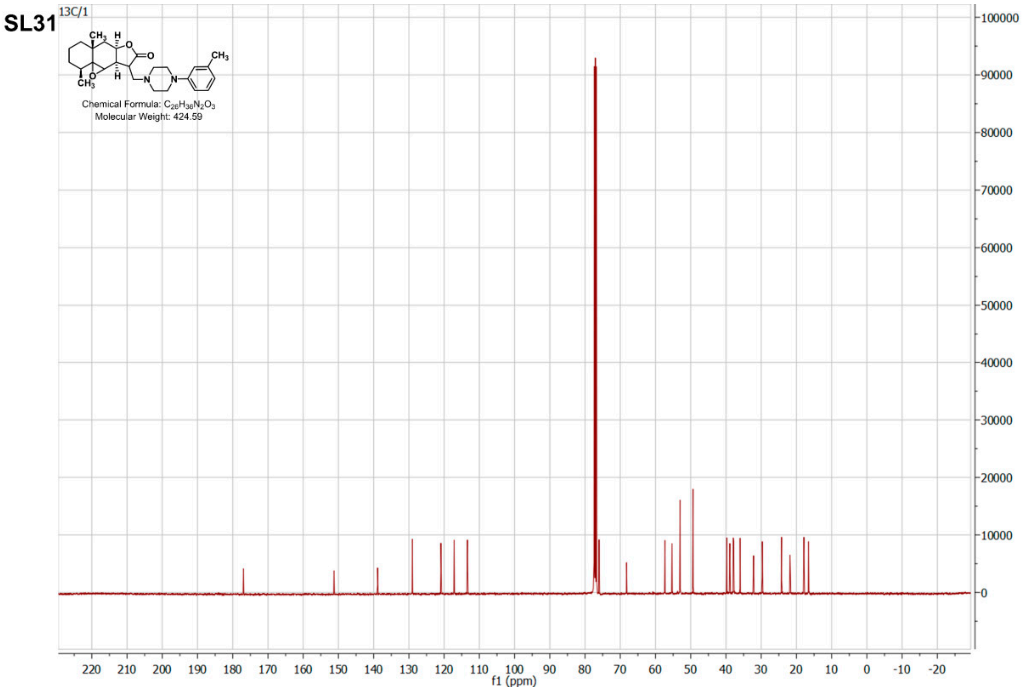

Figure S7. 13C NMR spectra of SL31

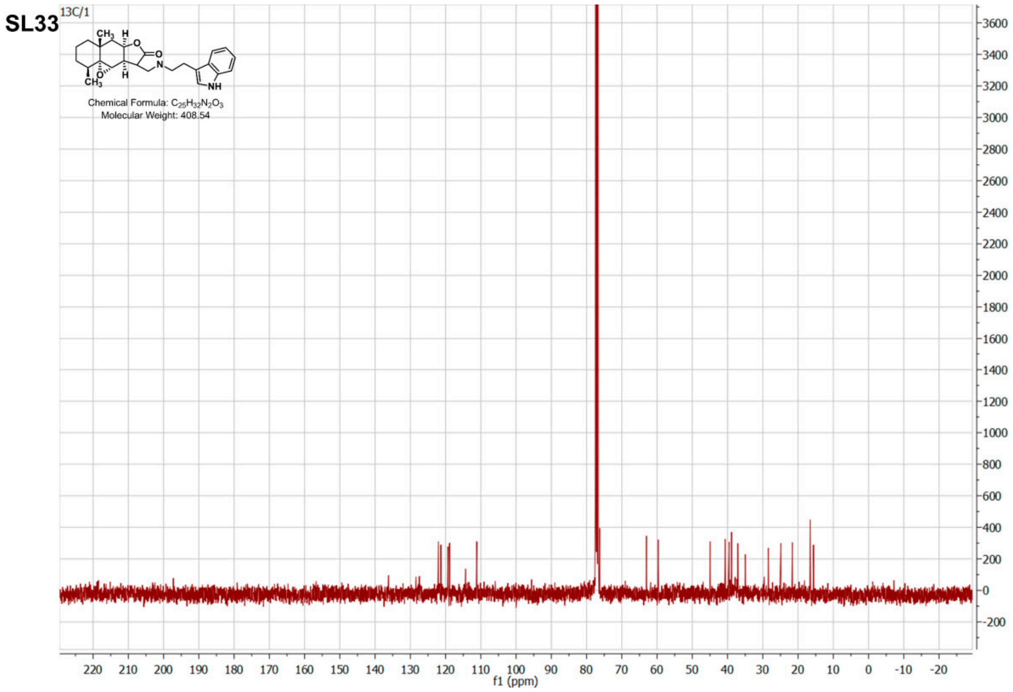

Figure S8. ^{13}C NMR spectra of SL33

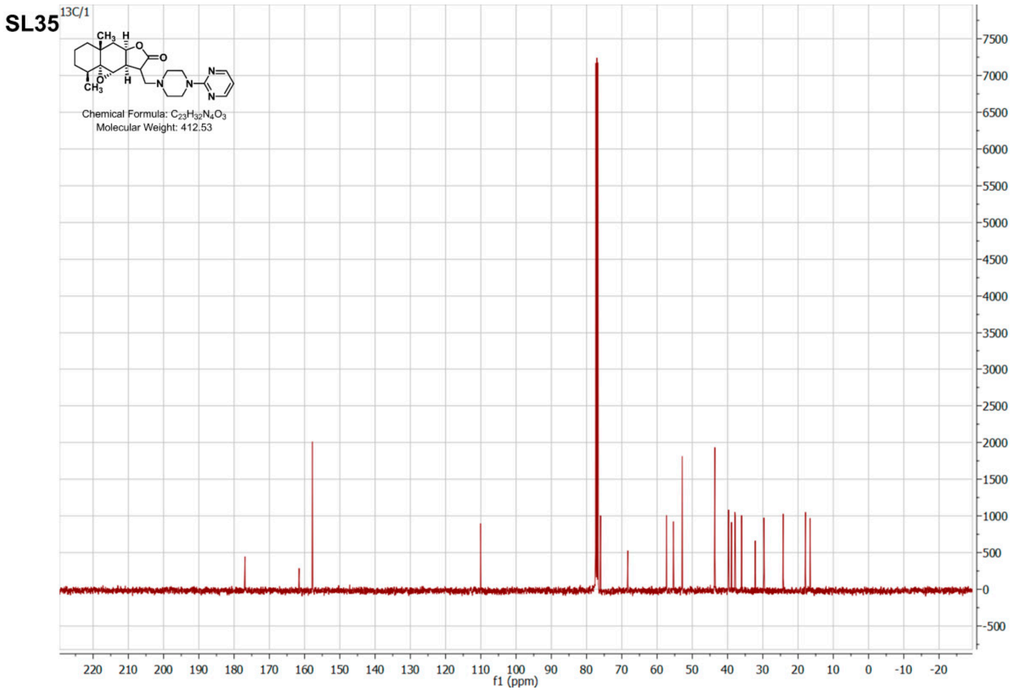

Figure S9. ^{13}C NMR spectra of SL35

SL8

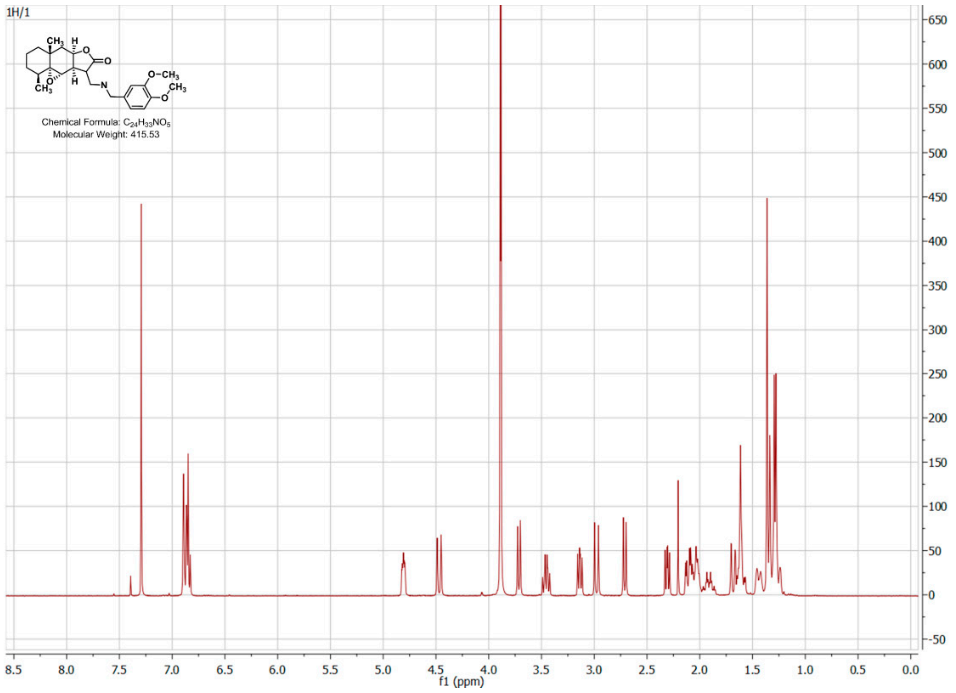

Figure S10. ^{1}H NMR spectra of SL8

SL10

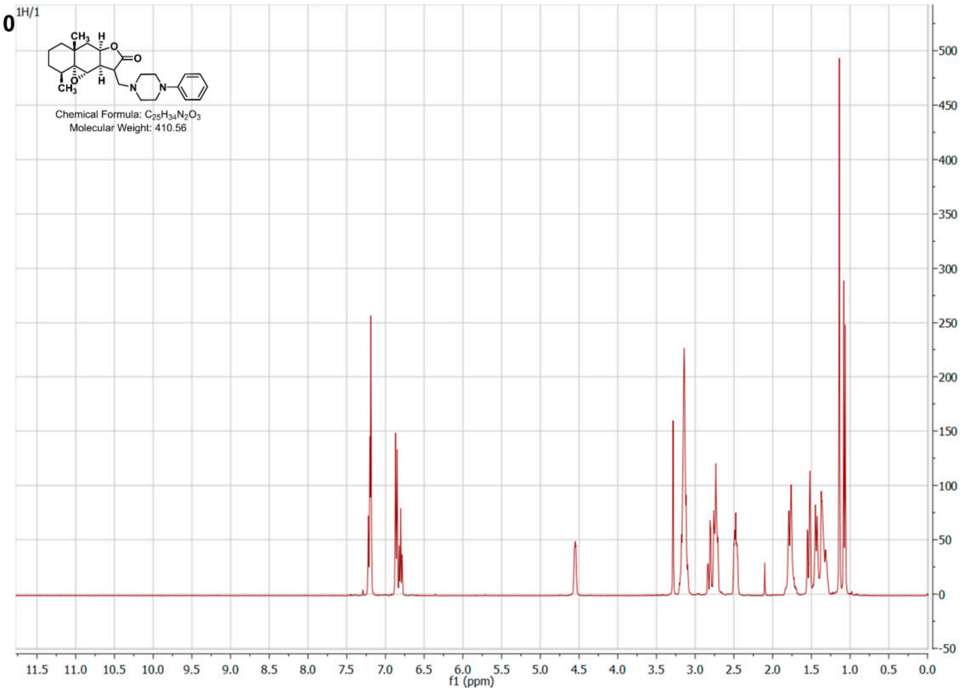

Figure S11. ^{1}H NMR spectra of SL10

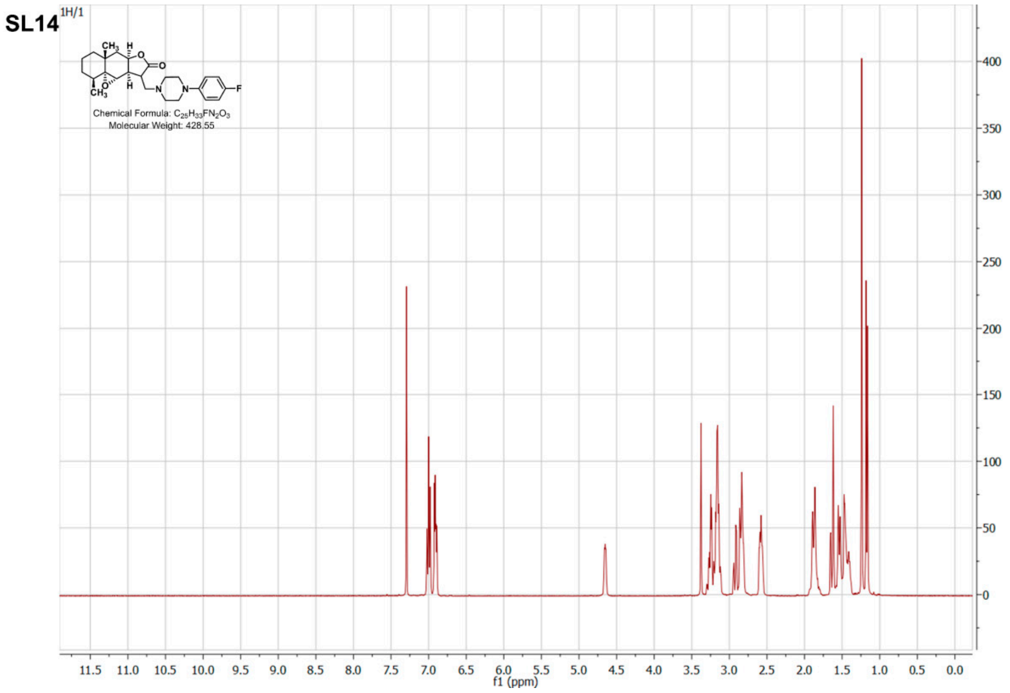

Figure S12. ^{1}H NMR spectra of SL14

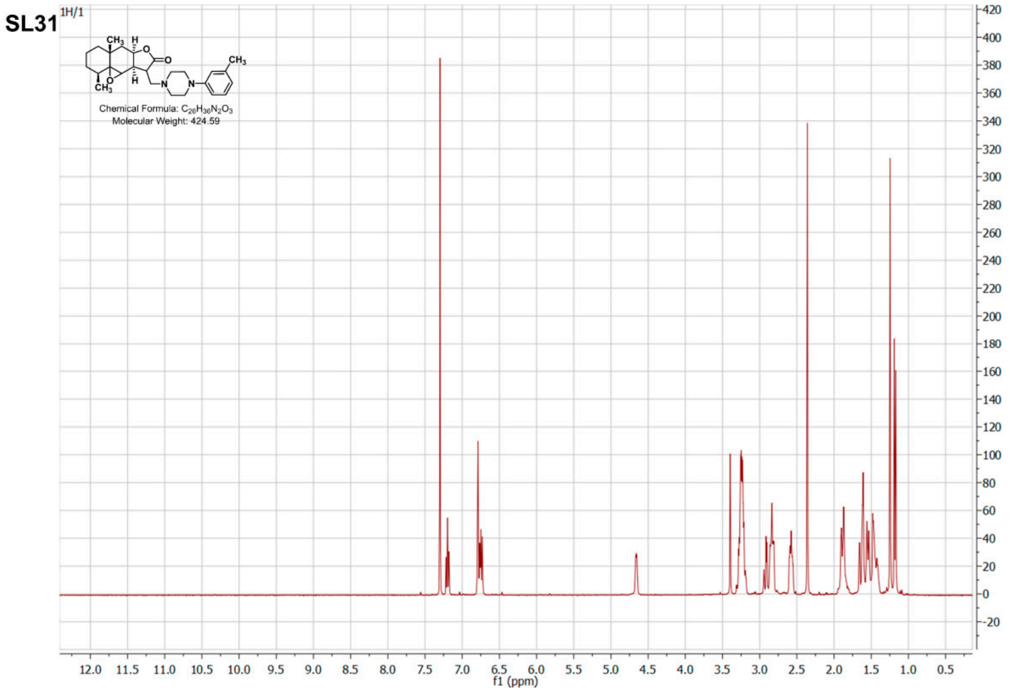

Figure S13. ^{1}H NMR spectra of SL31

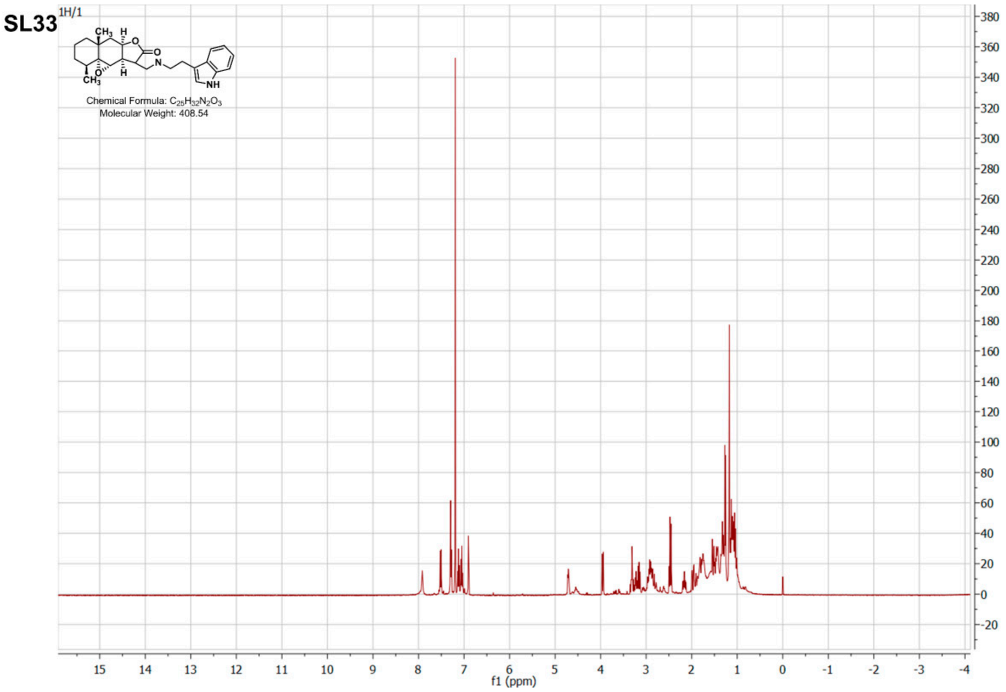

Figure S14. ^{1}H NMR spectra of SL33

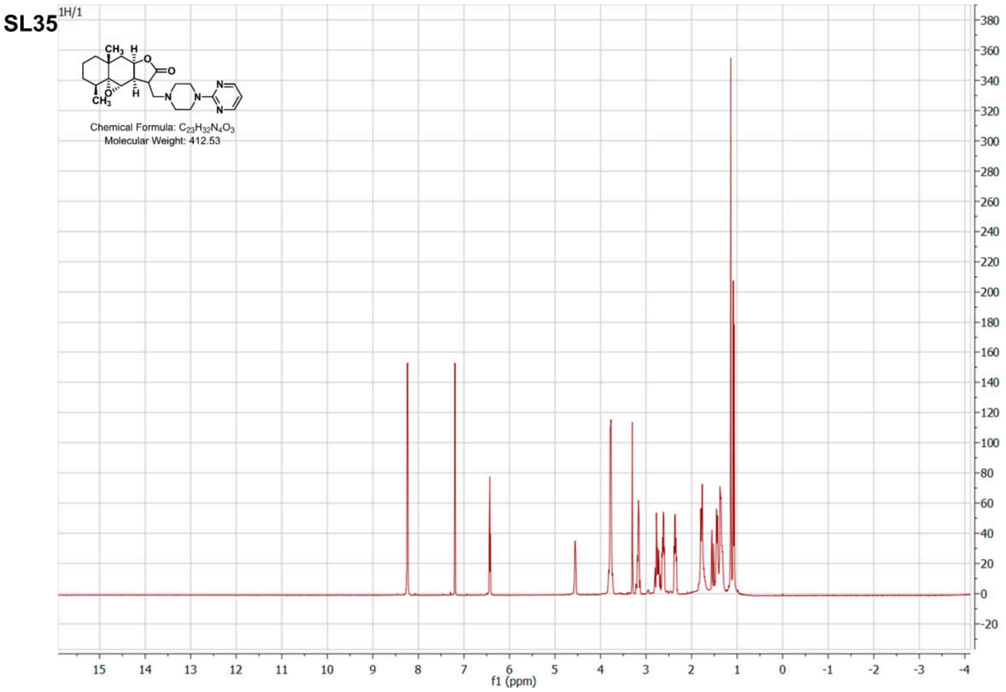

Figure S15. ^{1}H NMR spectra of SL35

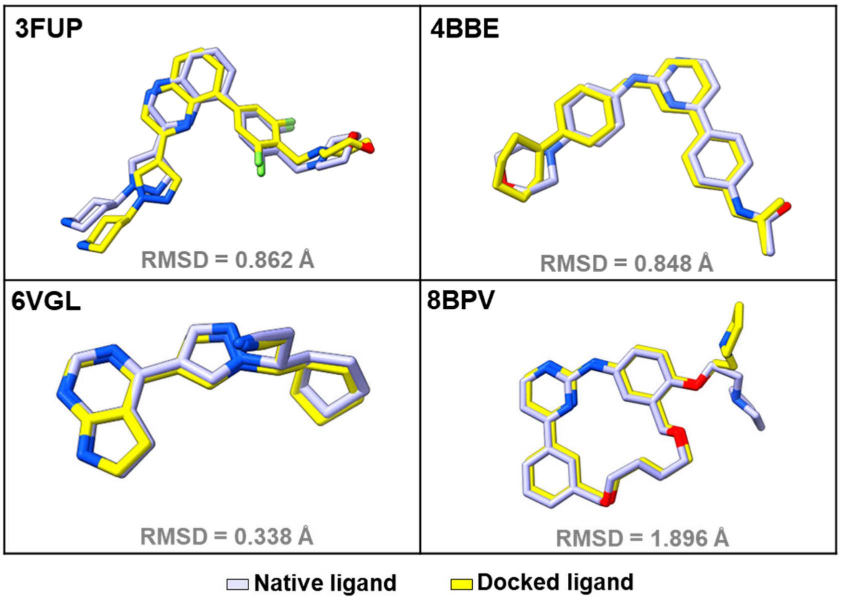

Figure S16. Docking validation of the GOLD protocol performed by redocking the native ligands into the corresponding JAK2 crystal structures (PDB IDs: 3FUP, 4BBE, 6VGL, and 8BPV). The superimposition of the crystallographic ligand (grey) and the predicted docked pose (yellow) is shown for each structure.
